# Curcumanes E and F, two rare sesquiterpenoids with a dicyclo[3.3.1]nonane moiety, from *Curcuma longa* and their vasorelaxant activities

**DOI:** 10.3389/fchem.2022.995950

**Published:** 2022-09-02

**Authors:** Juan Liu, Ming-Ming Qiao, Cheng Peng, Hong-Zhen Shu, Chun-Wang Meng, Fei Liu, Liang Xiong

**Affiliations:** ^1^ State Key Laboratory of Southwestern Chinese Medicine Resources, Chengdu University of Traditional Chinese Medicine, Chengdu, China; ^2^ School of Pharmacy, Chengdu University of Traditional Chinese Medicine, Chengdu, China; ^3^ Institute of Innovative Medicine Ingredients of Southwest Specialty Medicinal Materials, Chengdu University of Traditional Chinese Medicine, Chengdu, China

**Keywords:** *Curcuma long*a, Zingiberaceae, sesquiterpenoids, absolute configuration, vasorelaxant activity

## Abstract

Two new sesquiterpenoids, curcumanes E (**1**) and F (**2**), were isolated from the rhizome of *Curcuma longa*, and their structures and absolute configurations were examined using extensive spectroscopic analyses and ECD calculations. Interestingly, compounds **1** and **2** are diastereoisomers possessing a rare sesquiterpenoid skeleton that has been reported only once before. Both curcumanes E and F exhibit significant vasorelaxant effects against KCl-induced contraction of rat aortic rings, with EC_50_ values of 5.10 ± 0.79 and 5.58 ± 1.77 μM, respectively. These findings enrich the data concerning this rare type of sesquiterpenoids and further indicate that these rare sesquiterpenoids can effectively reduce blood pressure.

## Introduction

Sesquiterpenoids are representative terpenoid molecules that are widely distributed in plants, microbes, and microorganisms. Over 300 natural sesquiterpenoid skeletons have been reported so far (Cane, 1999; Liu et al., 2012), and novel skeletons are being discovered on a regular basis. The reported compounds have been found to have extensive bioactivities, such as anti-inflammatory (Chang et al., 2020), lipid regulatory (Zhu et al., 2020; Yin et al., 2021), antiviral (Liu et al., 2021), anti-proliferative (Wu et al., 2021), and proangiogenic (Han et al., 2022) activities. Thus, their intriguing structures and impressive bioactivities have attracted the attention of many chemists and pharmacologists.

**FIGURE 1:**
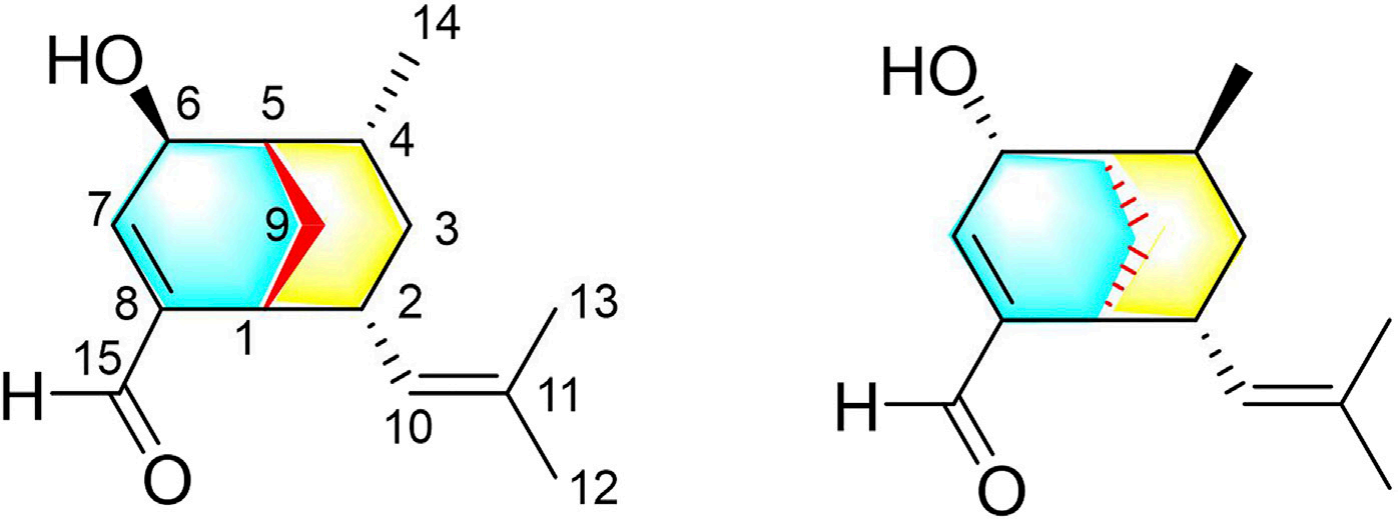
Structures of compounds **1** and **2**.

*Curcuma longa* L. (Zingiberaceae) is used as a traditional Chinese medicine (TCM) to promote blood circulation and remove blood stasis, and it is often added to food as a coloring and flavoring agent (Chen et al., 2010; Sun et al., 2017; Yuan et al., 2018). So far, more than 200 chemical components have been isolated from *C. longa*, including curcuminoids, sesquiterpenoids, monoterpenoids, and alkaloids (Singh et al., 2010; Xiao et al., 2011; Lin et al., 2012; Prete et al., 2016; Sun et al., 2017). These components exhibit a variety of pharmacological effects, including anti-inflammatory (Ti et al., 2021), antibacterial (Moghadamtousi et al., 2014), anticancer (Chen et al., 2014), and antioxidant (Llano et al., 2019) effects. As part of our long-term project to explore active natural compounds in blood-activating TCMs, we have continuously investigated the extract of the *C. longa* rhizome, and we have successfully isolated several novel sesquiterpenoids with significant vasorelaxant activity (Liu et al., 2019; Qiao et al., 2019; Chen et al., 2022). In particular, two bicyclic sesquiterpenoids (curcumanes A and B) possessing unprecedented skeletons with a dicyclo [3.2.1]octane or a dicyclo [3.3.1]nonane moiety have been isolated and identified (Liu et al., 2019). In addition, an unusual *seco*-cadinane sesquiterpenoid (curcumane C) and a pair of unusual *nor*-bisabolene enantiomers (curcumane D) with significant vasorelaxant activity have been isolated from *C. longa* (Qiao et al., 2019). To explore whether other rare sesquiterpenoids play a role in the vasorelaxant effect of *C. longa*, two curcumane B analogues (**1** and **2**) featuring a dicyclo [3.3.1]nonane moiety were isolated and characterized in this study (Figure 1). The isolation, structure elucidation, absolute configuration, and vasorelaxant activities of **1** and **2** are detailed hereafter.

## Experimental

### General experimental procedures

IR spectra and optical rotations were measured using an Agilent cary 600 FT-IR microscope (Agilent Technologies Inc., CA, United States) and an Anton Paar MCP 200 automatic polarimeter (Anton Paar GmbH, Austria), respectively. ECD spectra were recorded on an Applied photophysics Chirascan and Chirascan-plus circular dichroism spectrometer (Applied Photophysics Ltd., Leatherhead, England), while NMR spectra were recorded on a Bruker Avance III 600 NMR spectrometer (Bruker Corporation, Billerica, MA, United States) with solvent peaks as internal standards. HRESIMS measurements were carried out using a Q Exactive instrument (Thermo Scientific™, MA, United States), and TLC experiments were performed using glass plates precoated with silica gel (GF_254_, Qingdao Marine Chemical Inc., Qingdao, China). Silica gel (200–300 mesh, Yantai Institute of Chemical Technology, Yantai, China) and Sephadex LH-20 (Amersham Pharmacia Biotech AB, Uppsala, Sweden) were used for column chromatography. HPLC separations were achieved using an Agilent 1100 instrument (Agilent Technologies Inc., CA, United States) equipped with a Zorbax SB-C_18_ (250 × 9.4 mm^2^, 5 μm) semipreparative column. Vasorelaxant activity assays were conducted using a PL3508B6/C-V Panlab 8 Chamber Organ Bath System (including stimulating electrodes, Panlab eight-chamber organ baths, organ chambers, tissue hooks, and Labchart Pro software).

### Plant material

The rhizome of *Curcuma longa* L. (Zingiberaceae) was purchased from Sichuan Neautus Traditional Chinese Medicine Co., Ltd. (Chengdu, China) and identified by Prof. Min Li of Chengdu University of Traditional Chinese Medicine (Chengdu, China). A voucher specimen (CL-20160803) was deposited at the Institute of Innovative Medicine Ingredients of Southwest Specialty Medicinal Materials at Chengdu University of Traditional Chinese Medicine.

### Extraction and isolation

The dried rhizome of *C. longa* (50 kg) were extracted three times with 95% EtOH under reflux. The durations of the first, second, and third extractions were 3, 2, and 1.5 h, respectively. The yellow residue (7 kg) obtained by evaporating the EtOH extract under reduced pressure was dispersed in H_2_O and partitioned sequentially with petroleum ether and EtOAc. The EtOAc extract (3 kg) was separated on a silica gel column, using gradient elution with petroleum ether–EtOAc (1:0, 7:3, and 4:6) and EtOAc–MeOH (1:0, 1:1, and 0:1) to yield six fractions (A–F). Fraction B was separated further on a silica gel column using CH_2_Cl_2_–EtOAc (1:0–0:1) as eluent to yield 16 fractions (F_1_–F_16_). Subfractions F_6-1_–F_6-12_ were obtained from fraction F_6_ via RP-MPLC with gradient elution using a solution of MeOH in H_2_O (30–100%) as mobile phase. The F_6-7_ subfraction was further fractionated on a Sephadex LH-20 column (petroleum ether–CH_2_Cl_2_–MeOH, 5:5:1) to obtain subfractions F_6-7-1_ and F_6-7-2_. Finally, purification of F_6-7-1_ by preparative TLC (CH_2_Cl_2_–EtOAc, 15:1) and RP semipreparative HPLC (69% MeOH in H_2_O) afforded **1** (4.8 mg) and **2** (3.7 mg).

### Spectroscopic data

Curcumane E (**1**): colorless oil; ${\left[\text{α}\right]}_{D}^{25}$ +38.0 (*c* 0.16, MeOH); ECD (MeCN) *λ*_max_ (Δ*ε*) 190 (–32.7), 220 (+31.1), 248 (–11.9) nm, 339 (–4.4) nm; UV (MeCN) *λ*_max_ (log *ε*) 191 (3.78), 224 (3.44) nm; IR (ATR) *ν*_max_ 3389, 2919, 2865, 2720, 1686, 1523, 1450, 1381, 1256, 1162, 1004, 886, 827, 739, 679, 562 cm^−1^; ^1^H NMR (acetone-*d*_6_, 600 MHz) and ^13^C NMR (acetone-*d*_6_, 150 MHz) data, see Table 1. (+)-HRESIMS *m/z* 257.1504 [M + Na]^+^ (calcd for C_15_H_22_O_2_Na, 257.1512).

**Table 1:** 1H (600 MHz) and13C NMR (150 MHz) data of 1 and 2 (δ in ppm, J in Hz).

Position	1^a^		2^b^	
	*δ*_H_	*δ*_C_	*δ*_H_	*δ*_C_
1	2.81 m	31.1	2.65 q (3.0)	31.4
2	2.53 m	40.2	2.35 m	34.0
3a	1.24 dt (13.2, 4.2)	34.6	1.19 m	31.8
3b	0.70 q (13.2)		1.05 m	
4	1.71 m	35.2	1.94 m	29.3
5	1.91 m	43.0	1.94 m	42.6
6	4.21 d (3.6)	63.8	4.27 d (3.6)	63.8
7	6.90 brd (3.6)	152.9	6.82 dd (3.6, 1.2)	149.3
8		143.2		146.2
9a	1.75 dt (12.0, 3.6)	30.1	1.81 dt (13.2, 2.4)	23.6
9b	1.57 m		1.53 m	
10	4.53 brd (9.6)	129.0	5.46 brd (9.0)	125.6
11		130.8		133.1
12	1.55 brs	25.9	1.73 brs	26.1
13	1.63 brs	18.0	1.71 brs	18.0
14	1.00 d (7.2)	19.9	0.97 d (6.6)	20.0
15	9.49 s	193.8	9.50 s	193.6
OH	4.15 s			

a Data were measured in acetone-*d*_6_.

b Data were measured in CDCl_3_.

Curcumane F (**2**): colorless oil; ${\left[\text{α}\right]}_{D}^{25}$ –31.0 (*c* 0.09, MeOH); ECD (MeCN) *λ*_max_ (Δ*ε*) 214 (+2.6), 241 (–9.1) nm; UV (MeCN) *λ*_max_ (log *ε*) 194 (3.75), 221 (3.53) nm; IR (ATR) *ν*_max_ 3413, 2923, 2862, 2717, 1681, 1453, 1385, 1310, 1253, 1166, 1101, 1015, 961, 880, 660, 556cm^−1^; ^1^H NMR (CDCl_3_, 600 MHz) and ^13^C NMR (CDCl_3_, 150 MHz) data, see Table 1. (+)-HRESIMS *m/z* 257.1502 [M + Na]^+^ (calcd for C_15_H_22_O_2_Na, 257.1512).

### Effects of compounds 1 and 2 on the KCl-induced contractions of rat aortic rings

Male Sprague-Dawley rats (180–220 g) were purchased from Da Shuo Biotechnology Co., Ltd (Chengdu, Sichuan, China). All of the rats were housed under standard environmental conditions at a temperature between 25 ± 1°C, humidity between 50 ± 5%, and food and water were provided ad-libitum during the study period. All of the experimental procedures and protocols were approved by the Committee on the Ethics of Animal Experiments of Chengdu University of Traditional Chinese Medicine (Approval No. 2020–04) and followed the guidelines of the Management Committee for Experimental Animals, China.

The thoracic aorta of SD rats was carefully dissected and immediately immersed in 4°C oxygenated Krebs-Henseleit (K-H) solution [composition (mM): NaCl, 120; KCl, 4.6; KH_2_PO_4_, 1.2; MgSO_4_, 1.2; NaHCO_3_, 25; glucose, 10; CaCl_2_, 2.5]. Subsequently, 3–5-mm-long rings were prepared by cleaning the fat and connective tissues surrounding the aorta then cutting it. Before starting the experiment, the aortic rings were equilibrated in a 20 ml K-H solution (constant temperature of 37°C; bubbled with a gas mixture of 95% O_2_ and 5% CO_2_) for 1 h under 1 g initial tension. The aortic rings were stably pre-contracted by induction with 60 mM KCl solution, then cumulative concentrations of the test compounds (0.25, 0.75, 2.5, 7.5, and 25 μM) were added to the organ bath. All of the data were recorded using a computerized system, and Labchart Pro software was used to measure the tension of the prepared samples. Methoxyverapamil was used as a positive control (Xiong et al., 2015; Hu et al., 2018). The EC_50_ and *E*_max_ (maximal vasorelaxation) values of the test compounds and the positive drug were calculated based on the cumulative concentration–tension curves, and the relaxant responses of KCl-induced maximal contractile tension were considered 100%. Statistical analysis was performed using Student′s *t*-test, and *p* < 0.05 signified a statistically significant difference. All of the values are expressed as mean ± SEM.

## Results and discussion

### Structure elucidation of compounds

**FIGURE 2:**
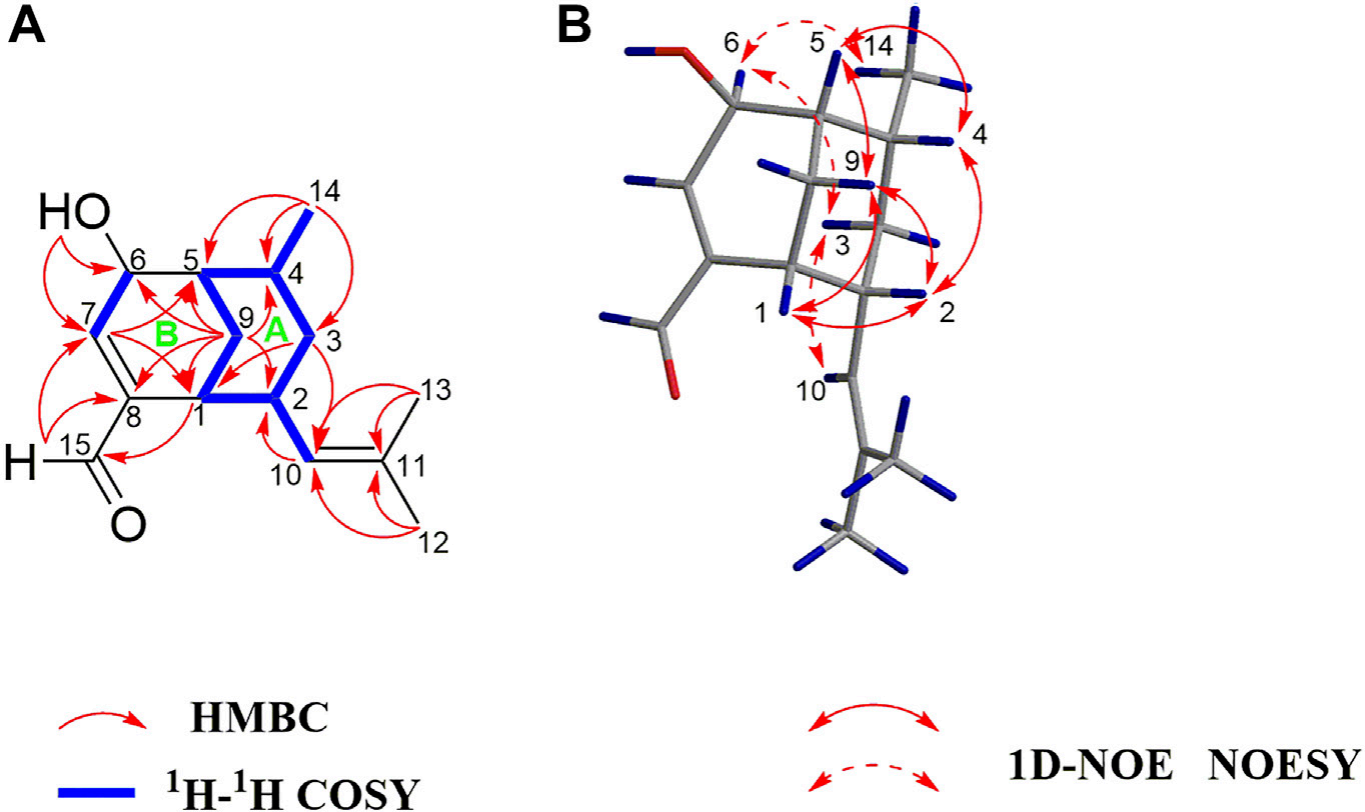
**(A)** Key ^1^H–^1^H COSY and HMBC correlations of **1**; **(B)** Key 1D-NOE and NOESY correlations of **1**.

Compound **1** was obtained as a colorless oil, and based on HRESIMS analysis, its molecular formula is C_15_H_22_O_2_ (*m/z* 257.1504 [M + Na]^+^; calcd 257.1512), which signifies that it has five degrees of unsaturation. The IR spectrum of **1** exhibits absorption bands corresponding to hydroxy (3389 cm^−1^), aldehyde (2865, 2720, and 1686 cm^−1^), and olefinic (1523 cm^−1^) groups. The resonance peaks observed in its ^1^H NMR spectrum may be attributed to three methyl groups [*δ*_H_ 1.00 (d, *J* = 7.2 Hz), 1.55 (brs), and 1.63 (brs)], two olefinic methines [*δ*_H_ 4.53 (brd, *J* = 9.6 Hz) and 6.90 (brd, *J* = 3.6 Hz)], an oxymethine [*δ*_H_ 4.21 (d, *J* = 3.6 Hz)], an aldehyde group [*δ*_H_ 9.49 (s)], and several aliphatic methylenes and methines between *δ*_H_ 0.70 and 2.81 (Table 1). The ^13^C NMR and DEPT data of **1** reveal the presence of one carbonylic carbon (*δ*_C_ 193.8), one oxygenated carbon [*δ*_C_ 63.8 (CH)], and four olefinic carbons [*δ*_C_ 129.0 (CH), 130.8 (C), 143.2 (C), 152.9 (CH)] (Table 1). Overall, the spectroscopic data indicate that compound **1** is a bicyclic sesquiterpenoid possessing an oxymethine group, an aldehyde group, and two trisubstituted double bonds. Based on the ^1^H−^1^H COSY correlations of H-1/H-2/H_2_-3/H-4/H-5/H_2_-9/H-1, H-2/H-10, and H-4/H_3_-14, as well as the HMBC correlations of H_3_-14 with C-3, C-4, and C-5; H_2_-3 with C-1, C-2, C-4, C-10, and C-14; H_2_-9 with C-1, C-2, C-4, and C-5; H-10 with C-1, C-2, C-3, C-12, and C-13; and H_3_-12 and H_3_-13 with C-10 and C-11 (Figure 2), compound **1** comprises a six-membered ring A with an isobutenyl unit at C-2 and a methyl group at C-4. The elucidation of the other six-membered ring B possessing OH-6 and CHO-8 substituents is based on the HMBC correlations of OH-6 with C-5, C-6, and C-7; H-15 with C-1, C-7, and C-8; H_2_-9 with C-6 and C-8; H-2 with C-8; and H-7 with C-1, C-5, and C-15. Thus, the planar structure of **1** is determined.

**FIGURE 3:**
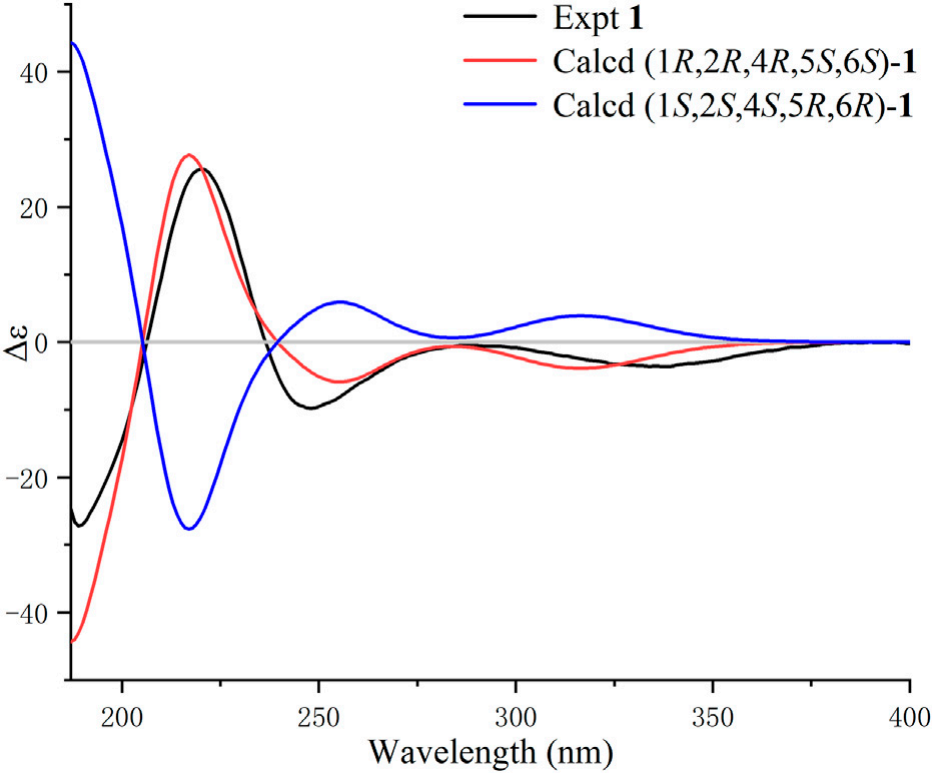
Calculated and experimental ECD spectra of **1** in MeCN.

The NOE difference spectrum of **1** shows that the H-2 and H-9b signals are enhanced when H-1 is irradiated; and the H-4 and H-9b signals are enhanced when H-5 is irradiated (Figure 2). This indicates that H-2 and H-4 have the same orientation as the methano bridge (C-1−C-9−C-5), which is consistent with the NOESY correlations of H-2 with H-4 and H-9b. However, the correlations of H_3_-14/H-6, H-3b/H-6, and H-3b/H-10 indicate that these protons are oriented in the opposite direction of the methano bridge. Based on the comparison of calculated and experimental ECD data, the absolute configuration of **1** is 1*R*,2*R*,4*R*,5*S*, 6*S* (Figure 3). Interestingly, compound **1** is an analogue of curcumane B, which was reported as a sesquiterpenoid with an unprecedented skeleton in 2019 (Liu et al., 2019). Compound **1** is labelled curcumane E.

**FIGURE 4:**
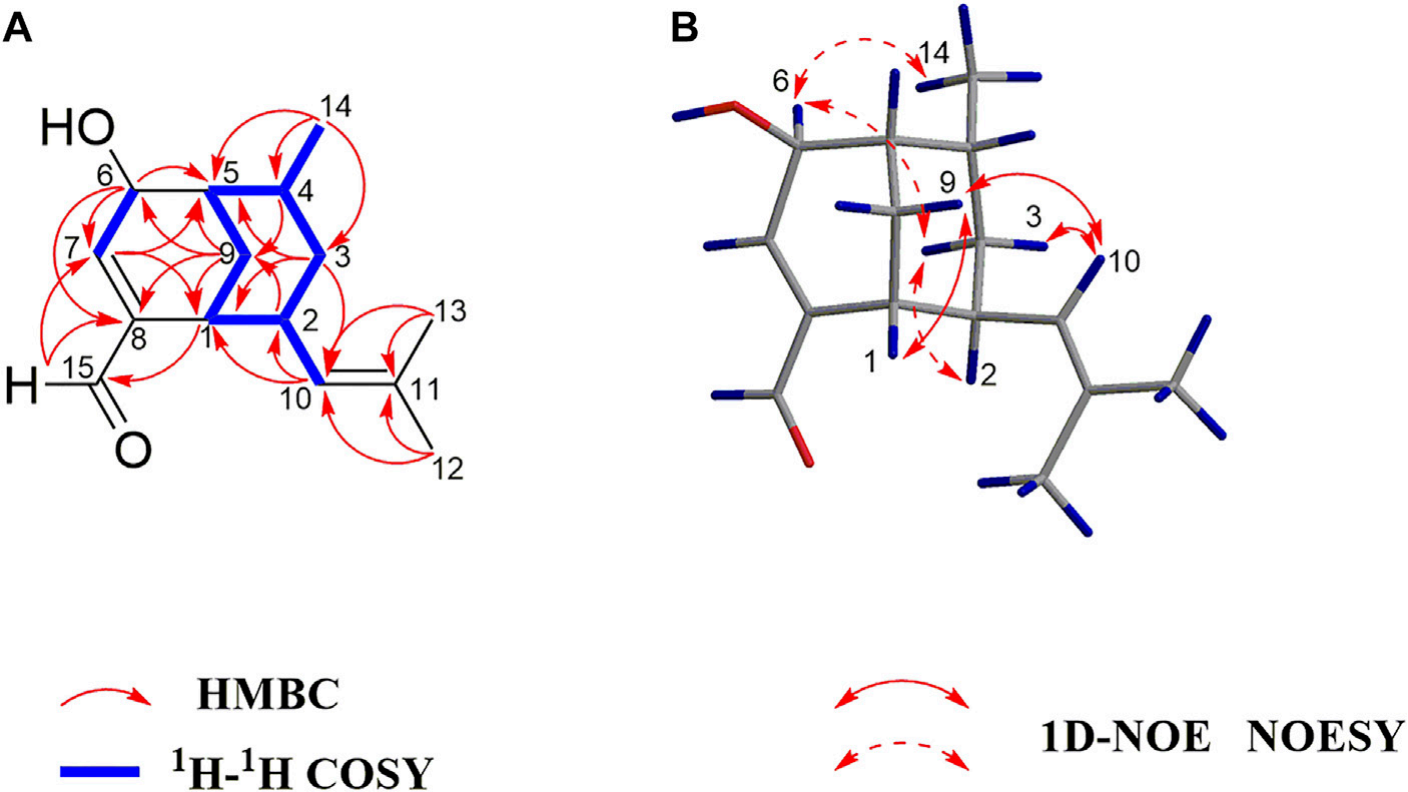
**(A)** Key ^1^H–^1^H COSY and HMBC correlations of **2**; **(B)** Key 1D-NOE and NOESY correlations of **2**.

**FIGURE 5:**
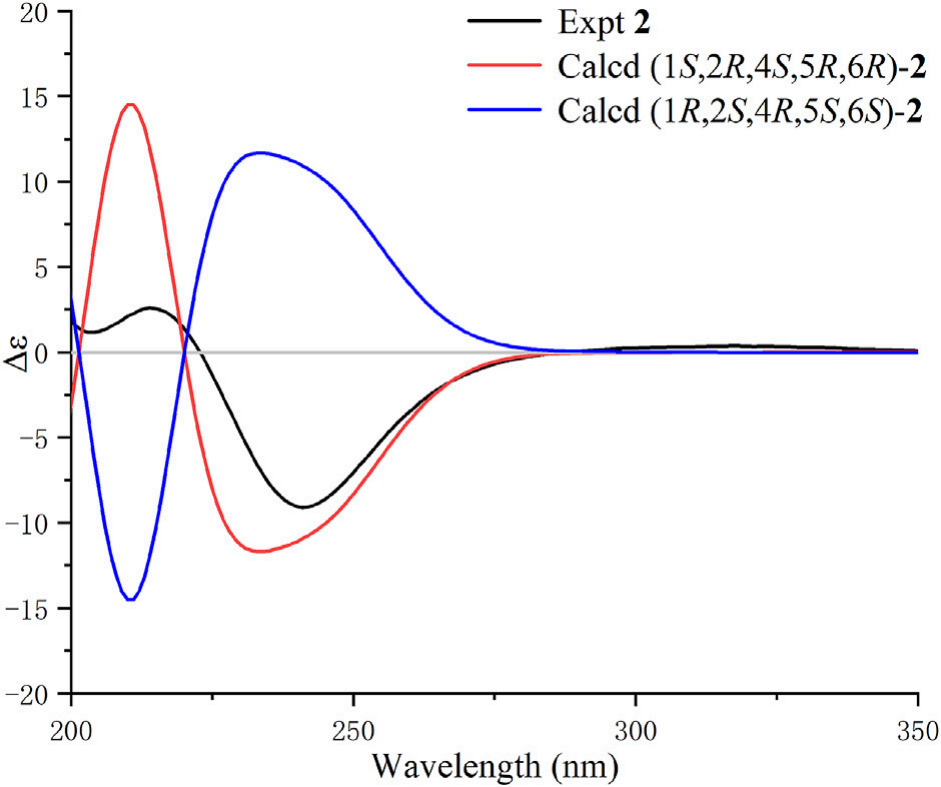
Calculated and experimental ECD spectra of **2** in MeCN.

The UV, IR, HRESIMS, and NMR data of compound **2** suggest that it is an isomer of compound **1**. Analysis of the 2D NMR (^1^H−^1^H COSY and HMBC) data of **2** (Figure 4) confirms that this compound has the same planar structure as **1**. However, the H-3b, H-4, H-10, and H-12 resonances in the ^1^H NMR spectrum of **2** are deshielded by Δ*δ*_H_ +0.35, +0.23, +0.93, and 0.18 ppm, respectively, compared to the same resonances in the spectrum of **1**. Meanwhile, the H-1 and H-2 resonances are shielded by Δ*δ*_H_ −0.16 and −0.18 ppm, respectively. The ^13^C chemical shifts of C-2, C-3, C-4, C-7, C-8, C-9, and C-10 in **2** are also different from those of the same carbon atoms in **1**. Therefore, compounds **2** and **1** are a pair of diastereomers. The NOESY spectrum of **2** exhibits correlations of H-6 with H_3_-14 and H-3b; and H-10 with H-9a and H-3a, which reveals that the isobutenyl-2, H-4, and OH-6 moieties are close to the methano bridges (C-1–C-9–C-5), while H-2, Me-4, and H-6 are oriented in the opposite direction of this bridge (Figure 4). Finally, the absolute configuration of **2** is elucidated as 1*S*,2*R*,4*S*,5*R*,6*R*, based on the comparison of the calculated and experimental ECD data (Figure 5). Compound **2** is labelled curcumane F.

### Effects of compounds 1 and 2 on the KCl-induced contractions of rat aortic rings

**FIGURE 6:**
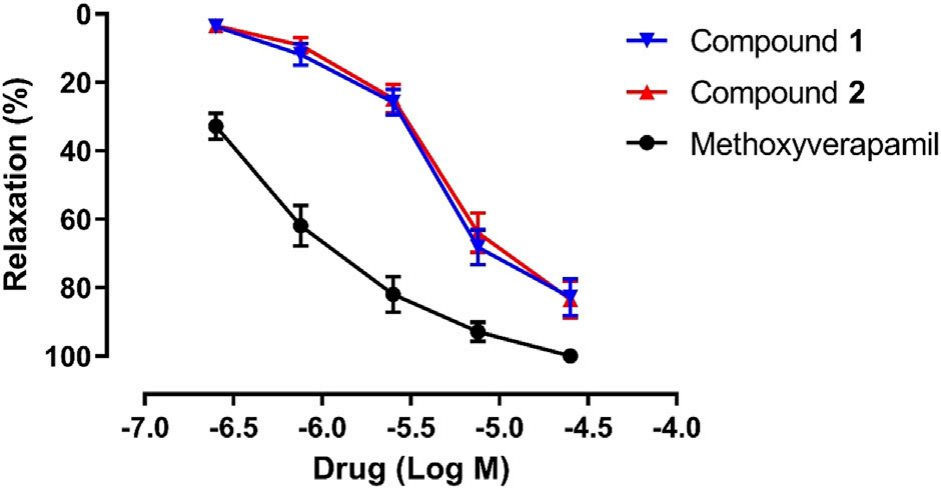
Vasorelaxant effects of compounds **1**, **2**, and methoxyverapamil on rat aortic rings pre-contracted with KCl (*n* = 5).

Previous studies show that the sesquiterpenoids isolated from *C. longa* possess endothelium-dependent vasorelaxant activity, endothelium-independent vasorelaxant activity, or both (Liu et al., 2019; Qiao et al., 2019; Chen et al., 2022). Therefore, the vasorelaxant effects of compounds **1** and **2** on pre-contracted rat aorta rings were investigated in this study, using methoxyverapamil as the positive control. As shown in Figure 6, compounds **1** and **2** exhibit a concentration-dependent relaxation effect on the KCl-induced contraction of rat aortic rings, with EC_50_ values of 5.10 ± 0.79 and 5.58 ± 1.77 μM, respectively (EC_50_ = 0.50 ± 0.05 μM for methoxyverapamil). The *E*_max_ values corresponding to the activities of **1**, **2**, and methoxyverapamil against KCl-induced contractions are 82.87 ± 5.36%, 83.44 ± 5.24%, and 100.00%, respectively. Unfortunately, no follow-up mechanism research was carried out due to the limited amounts of **1** and **2**. Notably, the vasorelaxant activities of **1** and **2** are similar, which suggests that this activity is not significantly affected by stereochemistry. However, a comparison of the EC_50_ values of compound **1** and curcumane B (Liu et al., 2019) indicates that the substituents at C-3 and C-8 play an important role in vasorelaxation.

## Conclusion

In summary, two unusual sesquiterpenoids with a dicyclo [3.3.1]nonane moiety (curcumanes E and F) were isolated from the rhizome of *C. longa*. The sesquiterpenoid skeleton characterized herein has been reported only once before (Liu et al., 2019). Moreover, curcumanes E and F are a pair of diastereomers that have similar vasorelaxant effects on the contracted rat aortic rings induced by KCl. Collectively, this study and our previous studies (Liu et al., 2019; Qiao et al., 2019) reveal that the rare sesquiterpenoids extracted from the rhizome of *C. longa* are considerably effective substances, even though they are not the main types of sesquiterpenoids in *C. longa*.

## Data Availability

The original contributions presented in the study are included in the article/Supplementary Material, further inquiries can be directed to the corresponding authors.
