# Supplementary material for: Curcumanes E and F, two rare sesquiterpenoids with a dicyclo[3.3.1]nonane moiety, from Curcuma longa and their vasorelaxant activities
Source: Front Chem. 2022 Sep 2;10:995950. doi: 10.3389/fchem.2022.995950 (PMC9478506; doi:10.3389/fchem.2022.995950)
Supplement: Supplementary file 1 [file DataSheet1.PDF]

# *Supplementary Material*

## The List of Contents


| No | Content | Page |
|---|---|---|
| 1 | **ECD calculation of compound 1** | 2 |
| 2 | **Figure S1.** *ω*B97XD/DGDZVP optimized 6 conformers of (1*R*,2*R*,4*R*,5*S*,6*S*)-**1** | 2 |
| 3 | **Table S1.** Energy analysis for the conformers of (1*R*,2*R*,4*R*,5*S*,6*S*)-**1** | 3 |
| 4 | **ECD calculation of compound 2** | 3 |
| 5 | **Figure S2.** *ω*B97XD/DGDZVP optimized 6 conformers of (1*S*,2*R*,4*S*,5*R*,6*R*)-**2** | 4 |
| 6 | **Table S2.** Energy analysis for the conformers of (1*S*,2*R*,4*S*,5*R*,6*R*)-**2** | 4 |
| 7 | **References** | 5 |
| 8 | **Figure S3.** The IR spectrum of compound **1** | 6 |
| 9 | **Figure S4.** The (+)-HRESIMS spectroscopic data of compound **1** | 7 |
| 10 | **Figure S5.** The $^{1}$H NMR spectrum of compound **1** in acetone-$d_6$ | 8 |
| 11 | **Figure S6.** The $^{13}$C NMR spectrum of compound **1** in acetone-$d_6$ | 9 |
| 12 | **Figure S7.** The DEPT spectrum of compound **1** in acetone-$d_6$ | 10 |
| 13 | **Figure S8.** The HSQC spectrum of compound **1** in acetone-$d_6$ | 11 |
| 14 | **Figure S9.** The $^{1}$H-$^{1}$H gCOSY spectrum of compound **1** in acetone-$d_6$ | 12 |
| 15 | **Figure S10.** The HMBC spectrum of compound **1** in acetone-$d_6$ | 13 |
| 16 | **Figure S11.** The NOESY spectrum of compound **1** in acetone-$d_6$ | 14 |
| 17 | **Figure S12.** The 1D-NOE spectrum of compound **1** in acetone-$d_6$ | 15 |
| 18 | **Figure S13.** The IR spectrum of compound **2** | 16 |
| 19 | **Figure S14.** The (+)-HRESIMS spectroscopic data of compound **2** | 17 |
| 20 | **Figure S15.** The $^{1}$H NMR spectrum of compound **2** in $CDCl_3$ | 18 |
| 21 | **Figure S16.** The $^{13}$C NMR spectrum of compound **2** in $CDCl_3$ | 19 |
| 22 | **Figure S17.** The DEPT spectrum of compound **2** in $CDCl_3$ | 20 |
| 23 | **Figure S18.** The HSQC spectrum of compound **2** in $CDCl_3$ | 21 |
| 24 | **Figure S19.** The $^{1}$H-$^{1}$H gCOSY spectrum of compound **2** in $CDCl_3$ | 22 |
| 25 | **Figure S20.** The HMBC spectrum of compound **2** in $CDCl_3$ | 23 |
| 26 | **Figure S21.** The NOESY spectrum of compound **2** in $CDCl_3$ | 24 |
| 27 | **Figure S22.** The 1D-NOE spectrum of compound **2** in $CDCl_3$ | 25 |

## ECD calculation of compound 1.

Conformation searches based on molecular mechanics with MMFF94s force field were performed for (1*R*,2*R*,4*R*,5*S*,6*S*)-**1** and gave 6 conformers (Boltzmann distribution ≥ 1%, Figure S1)[1]. The selected conformers were optimized using DFT at B3LYP/6-31G (d) level in vacuum with the Gaussian 16 program (Table S1) [2]. The B3LYP/6-31G (d)-optimized conformers (Boltzmann distribution ≥ 1%) were then reoptimized at the *ω*B97XD/DGDZVP level in acetonitrile. ECD computations for the *ω*B97XD/DGDZVP-optimized conformers (Boltzmann distribution ≥ 1%; Figure S1) were carried out at the CAM-B3LYP/DGDZVP level in acetonitrile[3]. Finally, according to the Boltzmann distribution theory and their relative Gibbs free energy ($\Delta G$), the ECD spectrum for (1*R*,2*R*,4*R*,5*S*,6*S*)-**1** was generated using SpecDis 1.71 with $\sigma = 0.30$ eV and UV shift +7 nm[4]. The corresponding theoretical ECD spectrum of (1*S*,2*S*,4*S*,5*R*,6*R*)-**1** was depicted by inverting that of (1*R*,2*R*,4*R*,5*S*,6*S*)-**1**. In the region of 190–400 nm, the theoretically calculated ECD spectrum of (1*R*,2*R*,4*R*,5*S*,6*S*)-**1** was agreed with the experimental ECD spectrum of **1**.

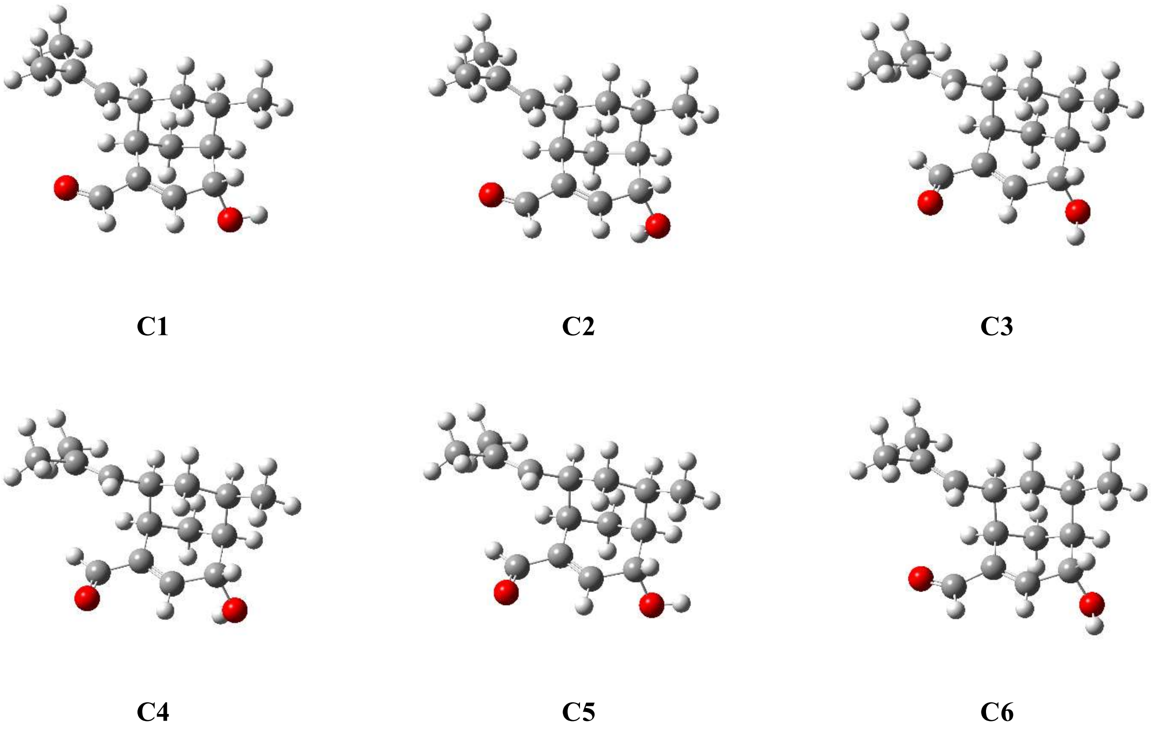


**Figure S1.** *ω*B97XD/DGDZVP optimized 6 conformers of (1*R*,2*R*,4*R*,5*S*,6*S*)-**1** (Boltzmann distribution ≥1%).

**Table S1.** Energy analysis for the conformers of (1*R*,2*R*,4*R*,5*S*,6*S*)-**1.**

| Conf. | MMFF energy | B3LYP/6-31G(d) Gibbs free energy (298.15 K) | | | *ω*B97XD/DGDZVP Gibbs free energy (298.15 K) | | |
|---|---|---|---|---|---|---|---|
| | ΔE (Kcal/ mol) | G (Hartree) | ΔG (Kcal/ mol) | Boltzmann Distribution | G (Hartree) | ΔG (Kcal/ mol) | Boltzmann Distribution |
| **C1** | 0.00 | -734.955752 | 0.0000 | 0.089 | -734.818324 | 0.0000 | 0.216 |
| **C2** | 0.0391 | -734.957306 | -0.9750 | 0.460 | -734.819127 | -0.5040 | 0.507 |
| **C3** | 0.4485 | -734.954086 | 1.0450 | 0.015 | -734.81618 | 1.3450 | 0.022 |
| **C4** | 0.8668 | -734.954981 | 0.4840 | 0.039 | -734.8168 | 0.9560 | 0.043 |
| **C5** | 1.0998 | -734.953349 | 1.5080 | 0.007 | -734.816308 | 1.2650 | 0.026 |
| **C6** | 1.1153 | -734.955568 | 0.1150 | 0.073 | -734.818183 | 0.0880 | 0.186 |

**ECD calculation of compound 2.**

Conformation searches based on molecular mechanics with MMFF94s force field were performed for (1*S*,2*R*,4*S*,5*R*,6*R)*-**2** and gave 6 conformers[1]. The selected conformers were optimized using DFT at B3LYP/6-31G (d) level in vacuum with the Gaussian 16 program (Table S2)[2]. The B3LYP/6-31G (d)-optimized conformers (Boltzmann distribution ≥ 1%) were then reoptimized at the *ω*B97XD/DGDZVP level in acetonitrile. ECD computations for the *ω*B97XD/DGDZVP-optimized conformers (Boltzmann distribution ≥ 1%; Figure S2) were carried out at the CAM-B3LYP/DGDZVP level in acetonitrile[3]. Finally, according to the Boltzmann distribution theory and their relative Gibbs free energy (ΔG), the ECD spectrum for (1*S*,2*R*,4*S*,5*R*,6*R)*-**2** was generated using SpecDis 1.71 with σ = 0.35 eV and UV shift +5 nm[4]. The corresponding theoretical ECD spectrum of (1*R*,2*S*,4*R*,5*S*,6*S)*-**2** was depicted by inverting that of (1*S*,2*R*,4*S*,5*R*,6*R)*-**2**.

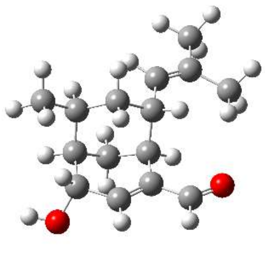

**C1**

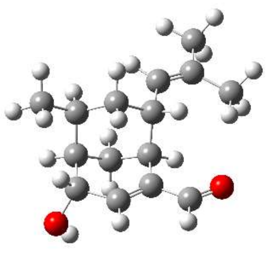

**C2**

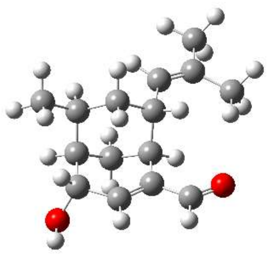

**C3**

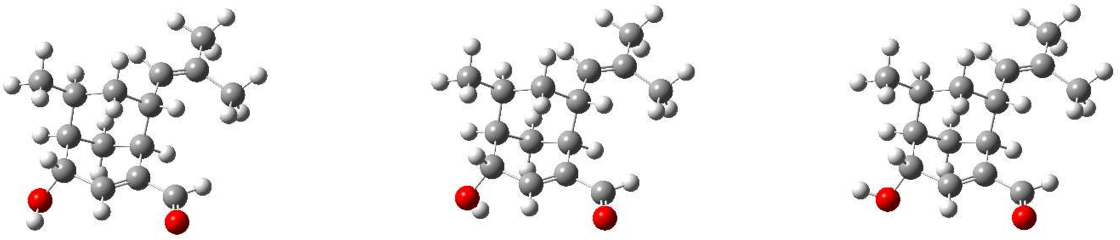

**C4** **C5** **C6**

**Figure S2.** *ω*B97XD/DGDZVP optimized 6 conformers of (1*S*,2*R*,4*S*,5*R*,6*R*)-**2** (Boltzmann distribution ≥1%).

**Table S2.** Energy analysis for the conformers of (1*S*,2*R*,4*S*,5*R*,6*R*)-**2**.

| Conf. | MMFF energy | B3LYP/6-31G(d) Gibbs free energy (298.15 K) | | | *ω*B97XD/DGDZVP Gibbs free energy (298.15 K) | | |
|---|---|---|---|---|---|---|---|
| | ΔE (Kcal/mol) | G (Hartree) | ΔG (Kcal/mol) | Boltzmann Distribution | G (Hartree) | ΔG (Kcal/mol) | Boltzmann Distribution |
| **C1** | 0.00 | -734.955446 | 0.0000 | 0.154 | -734.817687 | 0.0000 | 0.264 |
| **C2** | 0.0965 | -734.956901 | -0.9130 | 0.717 | -734.818293 | -0.3800 | 0.502 |
| **C3** | 1.1373 | -734.955186 | 0.1630 | 0.117 | -734.817492 | 0.1220 | 0.215 |
| **C4** | 3.0411 | -734.951739 | 2.3260 | 0.003 | -734.813762 | 2.4630 | 0.004 |
| **C5** | 3.3749 | -734.95265 | 1.7550 | 0.008 | -734.81463 | 1.9180 | 0.01 |
| **C6** | 3.6265 | -734.951068 | 2.7470 | 0.001 | -734.813908 | 2.3710 | 0.005 |

**References**

(1) (a) Goto, H.; Osawa, E. Corner flapping: a simple and fast algorithm for exhaustive generation of ring conformations. *J. Am. Chem. Soc.* 1989, 111, 8950−8951. (b) Goto, H.; Osawa, E. An efficient algorithm for searching low-energy conformers of cyclic and acyclic molecules. *J. Chem. Soc., Perkin Trans. 2* 1993, 2, 187−198.

(2) Frisch, M. J.; Trucks, G. W.; Schlegel, H. B.; Scuseria, G. E.; Robb, M. A.; Cheeseman, J. R.; Scalmani, G.; Barone, V.; Petersson, G. A.; Nakatsuji, H.; Li, X.; Caricato, M.; Marenich, A. V.; Bloino, J.; Janesko, B. G.; Gomperts, R.; Mennucci, B.; Hratchian, H. P.; Ortiz, J. V.; Izmaylov, A. F.; Sonnenberg, J. L.; Williams-Young, D.; Ding, F.; Lipparini, F.; Egidi, F.; Goings, J.; Peng, B.; Petrone, A.; Henderson, T.; Ranasinghe, D.; Zakrzewski, V. G.; Gao, J.; Rega, N.; Zheng, G.; Liang, W.; Hada, M.; Ehara, M.; Toyota, K.; Fukuda, R.; Hasegawa, J.; Ishida, M.; Nakajima, T.; Honda, Y.; Kitao, O.; Nakai, H.; Vreven, T.; Throssell, K.; Montgomery, J. A. Jr.; Peralta, J. E.; Ogliaro, F.; Bearpark, M. J.; Heyd, J.J.; Brothers, E. N.; Kudin, K. N.; Staroverov, V.N.; Keith, T. A.; Kobayashi, R.; Normand, J.; Raghavachari, K.; Rendell, A. P.; Burant, J. C.; Iyengar, S. S.; Tomasi, J.; Cossi, M.; Millam, J. M.; Klene, M.; Adamo, C.; Cammi, R.; Ochterski, J. W.; Martin, R. L.; Morokuma, K.; Farkas, O.; Foresman, J. B.; Fox, D. J. Gaussian 16, Revision B.01, Gaussian, Inc., Wallingford CT, 2016.

(3) Liu, Y.; Liu, F.; Qiao M. M.; Guo, L.; Chen, M. H.; Peng, C.; Xiong, L. Curcumanes A and B, Two Bicyclic Sesquiterpenoids with Significant Vasorelaxant Activity from *Curcuma longa*. *Org. Lett.* 2019, 21(4), 1197−1201.

(4) Bruhn, T.; Schaumlöffel, A.; Hemberger, Y.; Bringmann, G. Spec Dis, version 1.71, University of Würzburg, Germany, 2017.

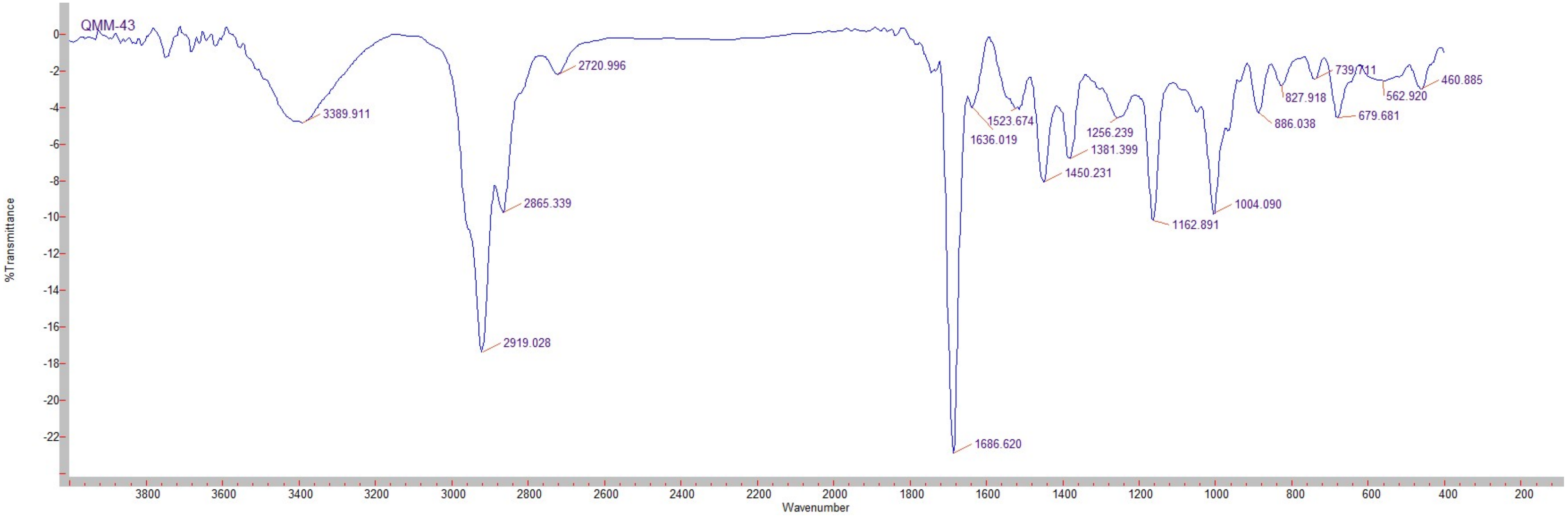


**Figure S3.** The IR spectrum of compound **1**

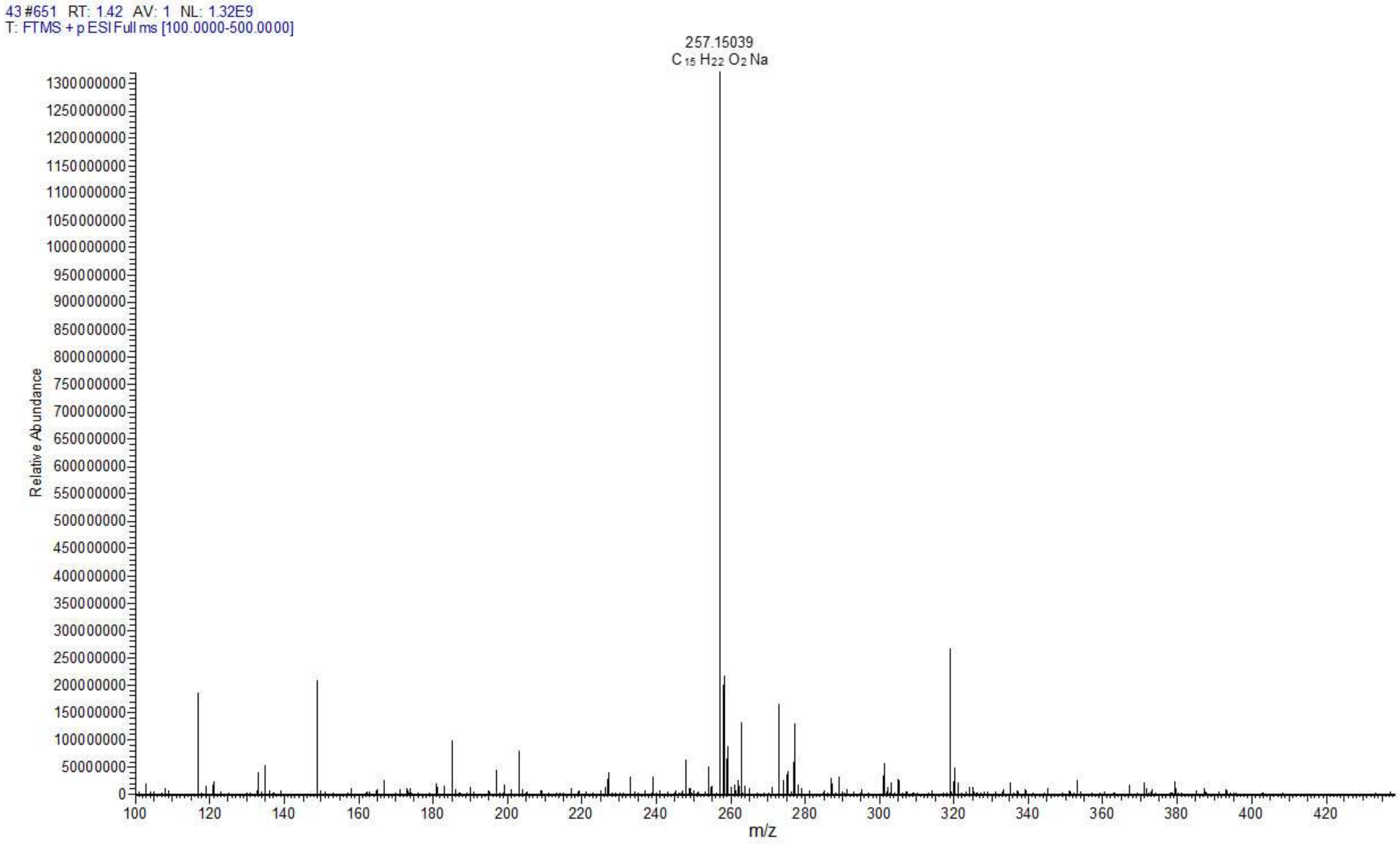


**Figure S4.** The (+)-HRESIMS spectroscopic data of compound **1**

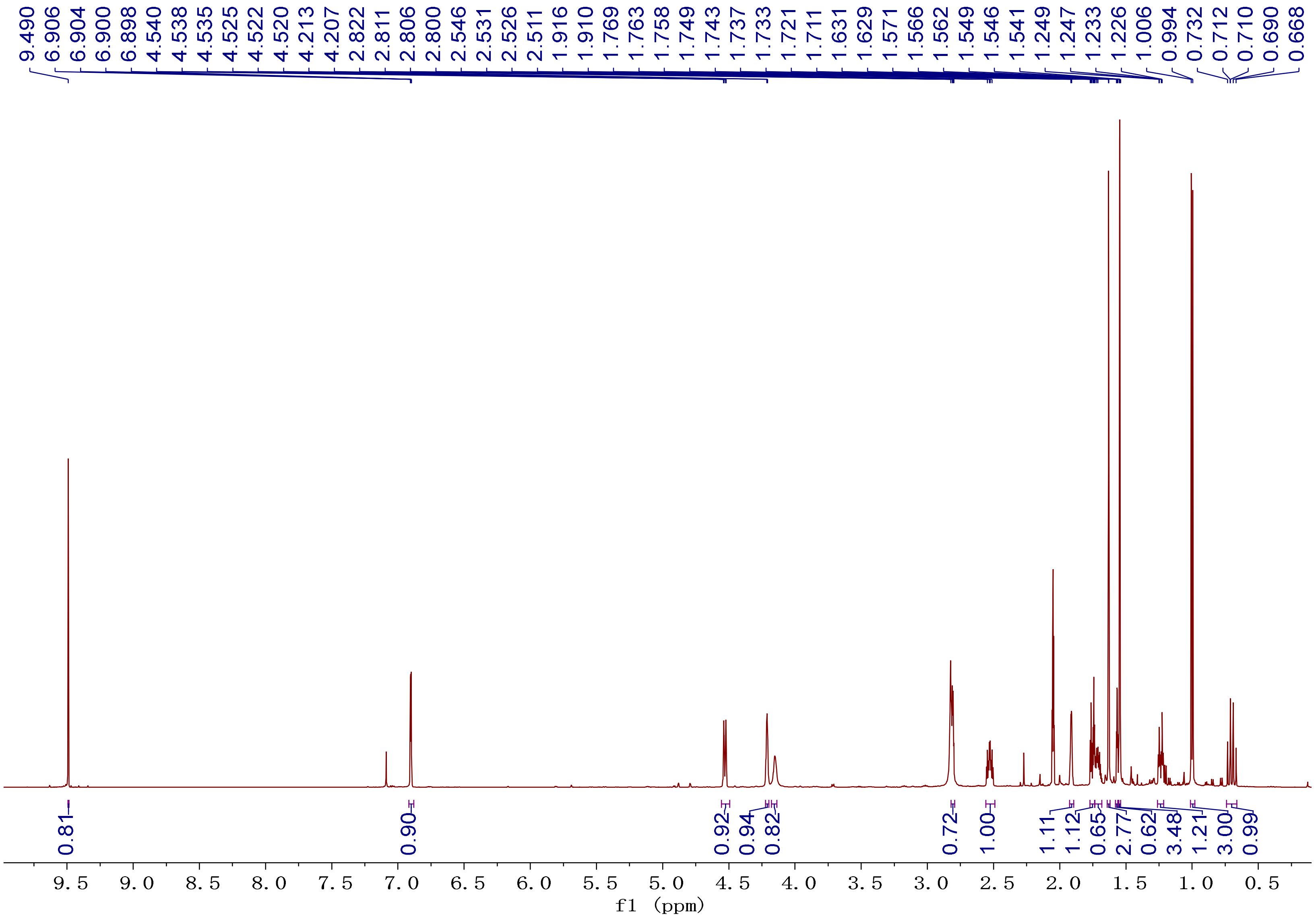


**Figure S5.** The $^{1}$H NMR spectrum of compound **1** in acetone-$d_6$

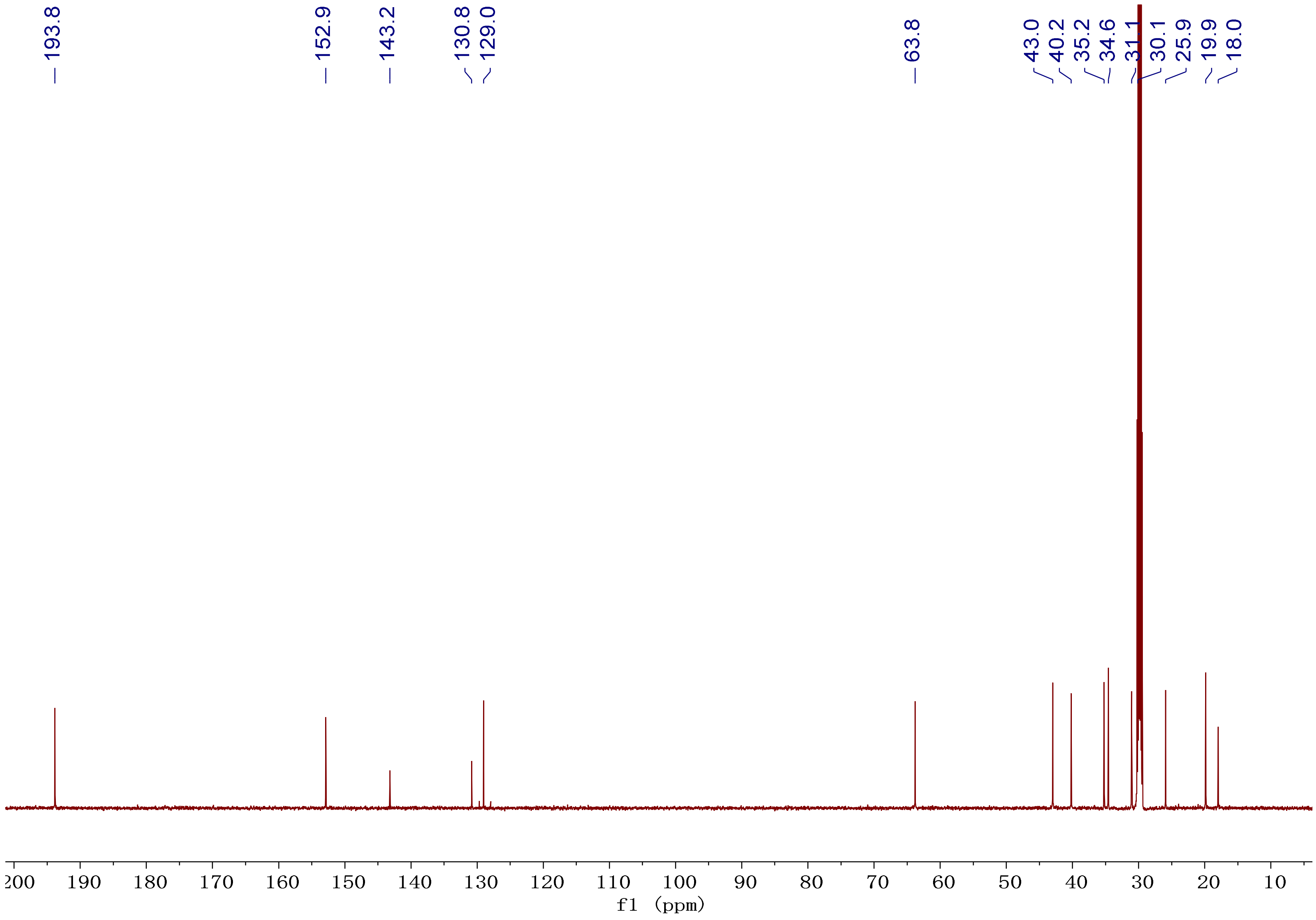


**Figure S6.** The $^{13}$C NMR spectrum of compound **1** in acetone-$d_6$

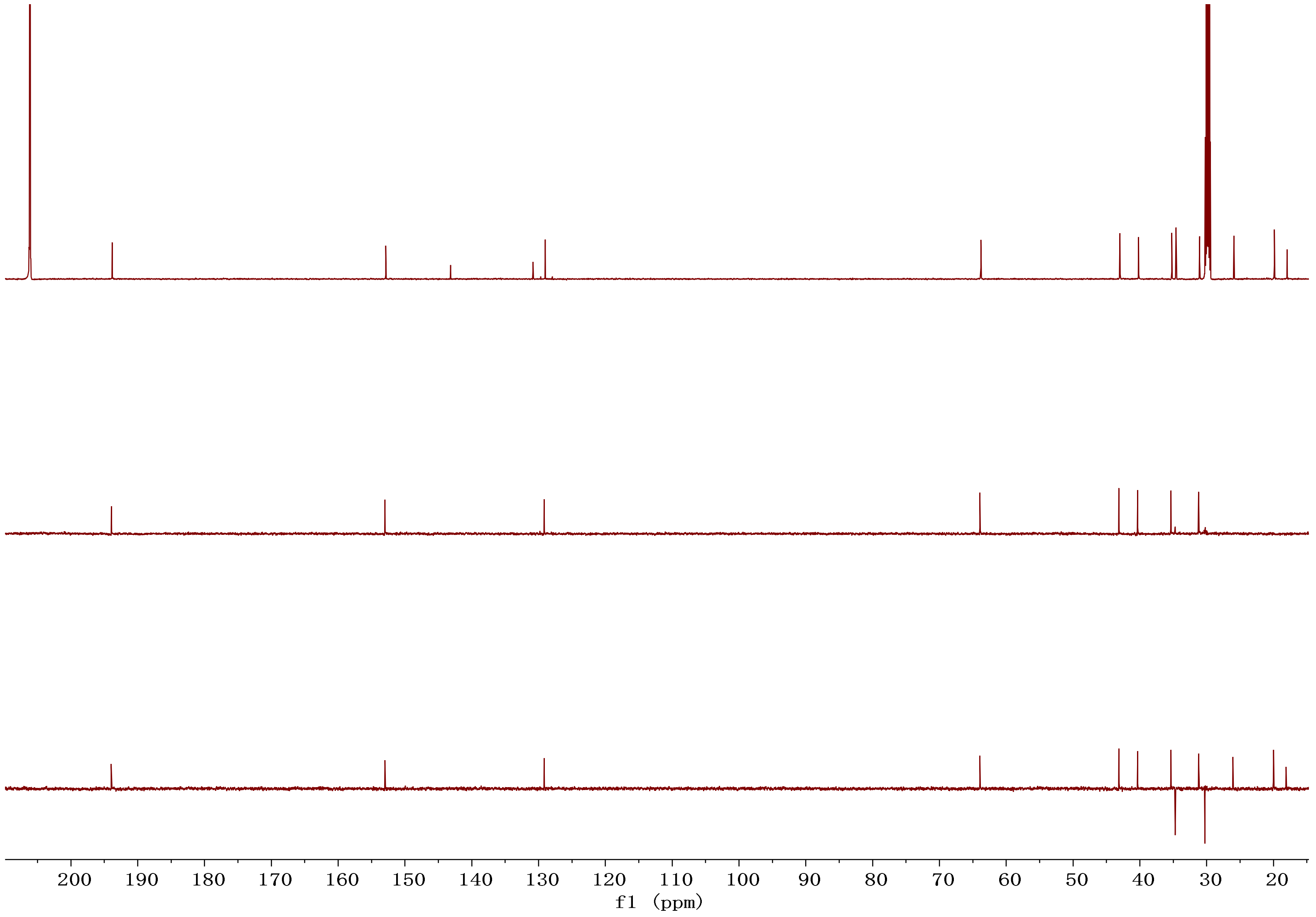


**Figure S7.** The DEPT spectrum of compound **1** in acetone-$d_6$

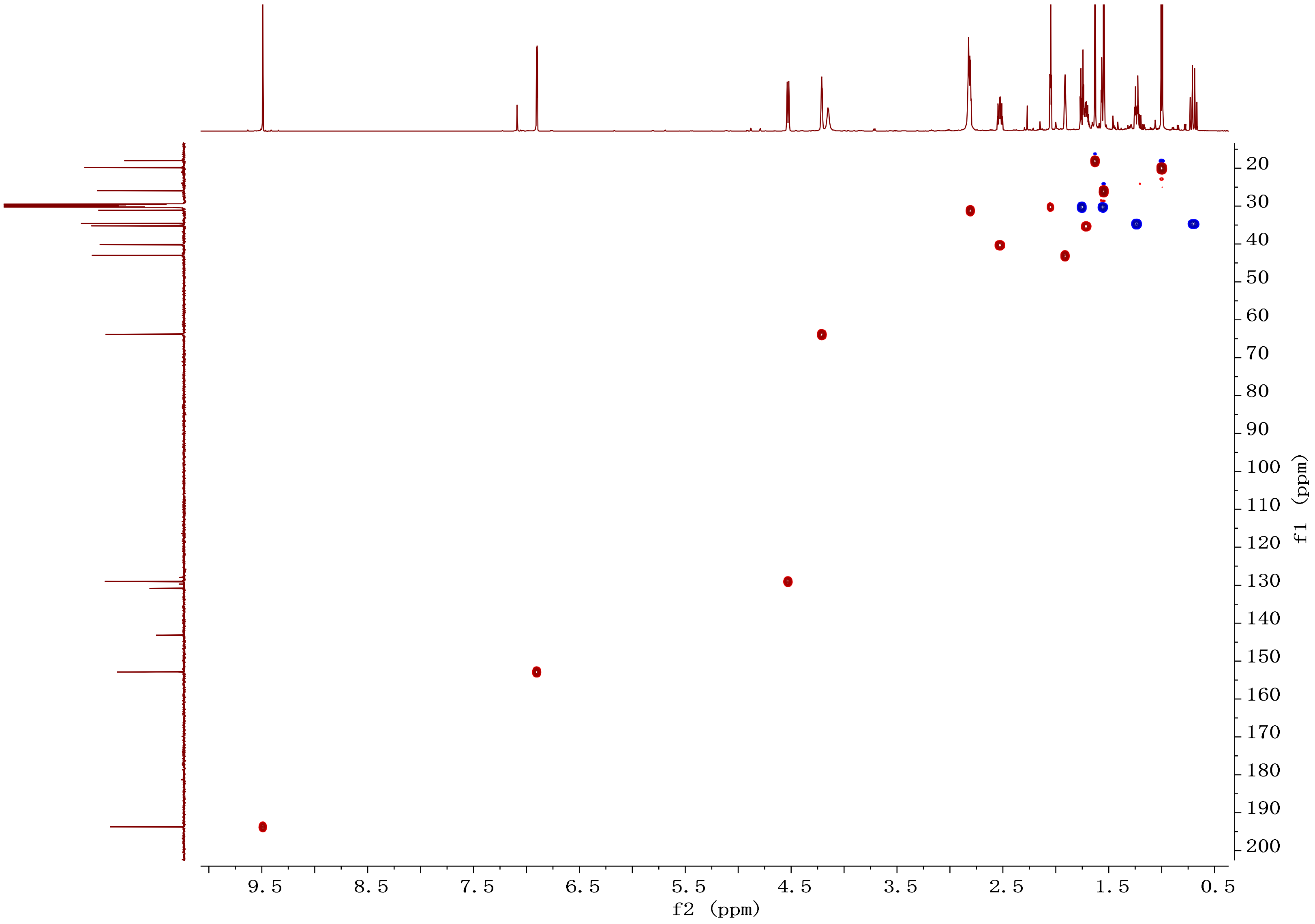


**Figure S8.** The HSQC spectrum of compound **1** in acetone-$d_6$

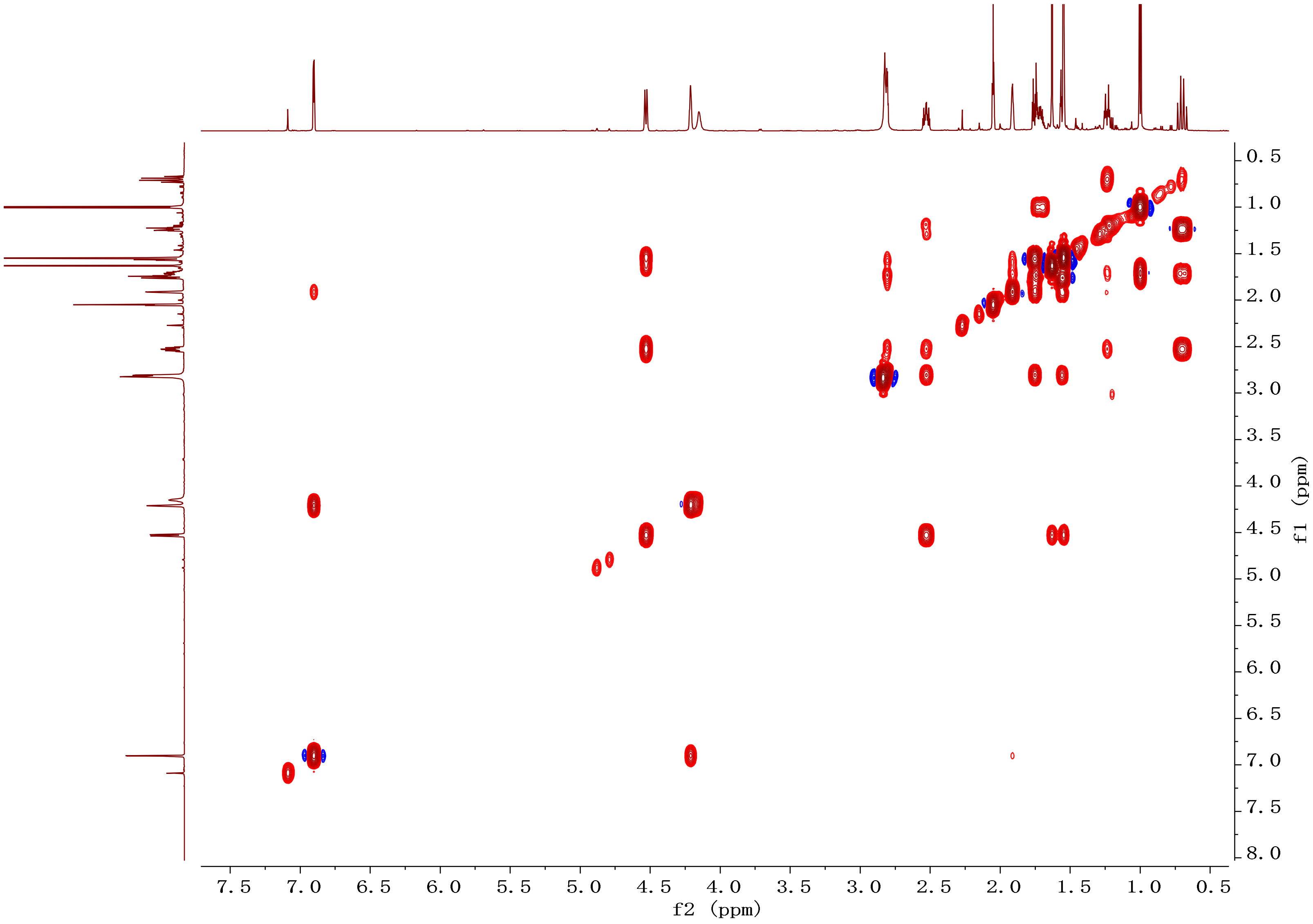


**Figure S9.** The $^{1}$H-$^{1}$H gCOSY spectrum of compound **1** in acetone-$d_6$

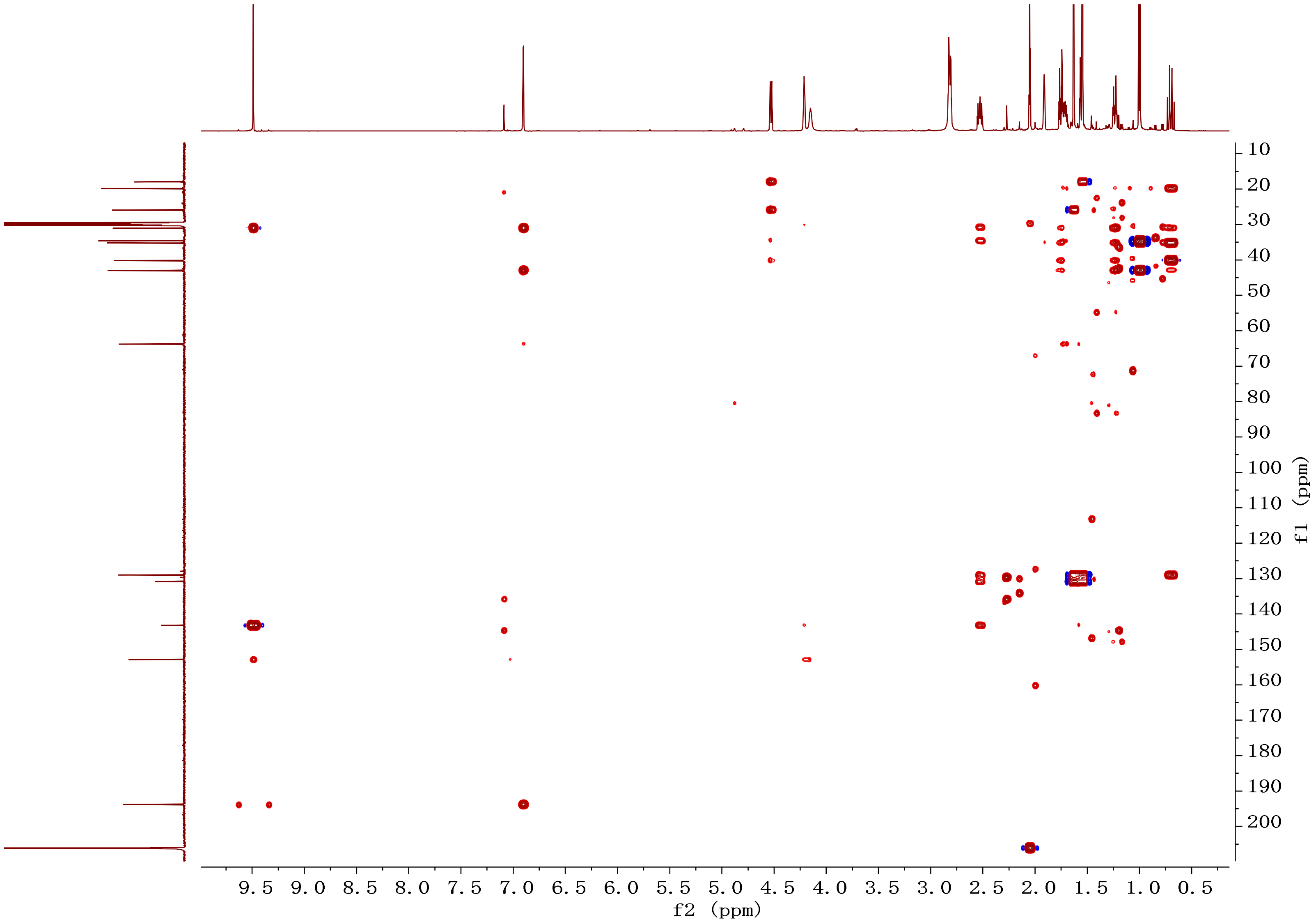


**Figure S10.** The HMBC spectrum of compound **1** in acetone-$d_6$

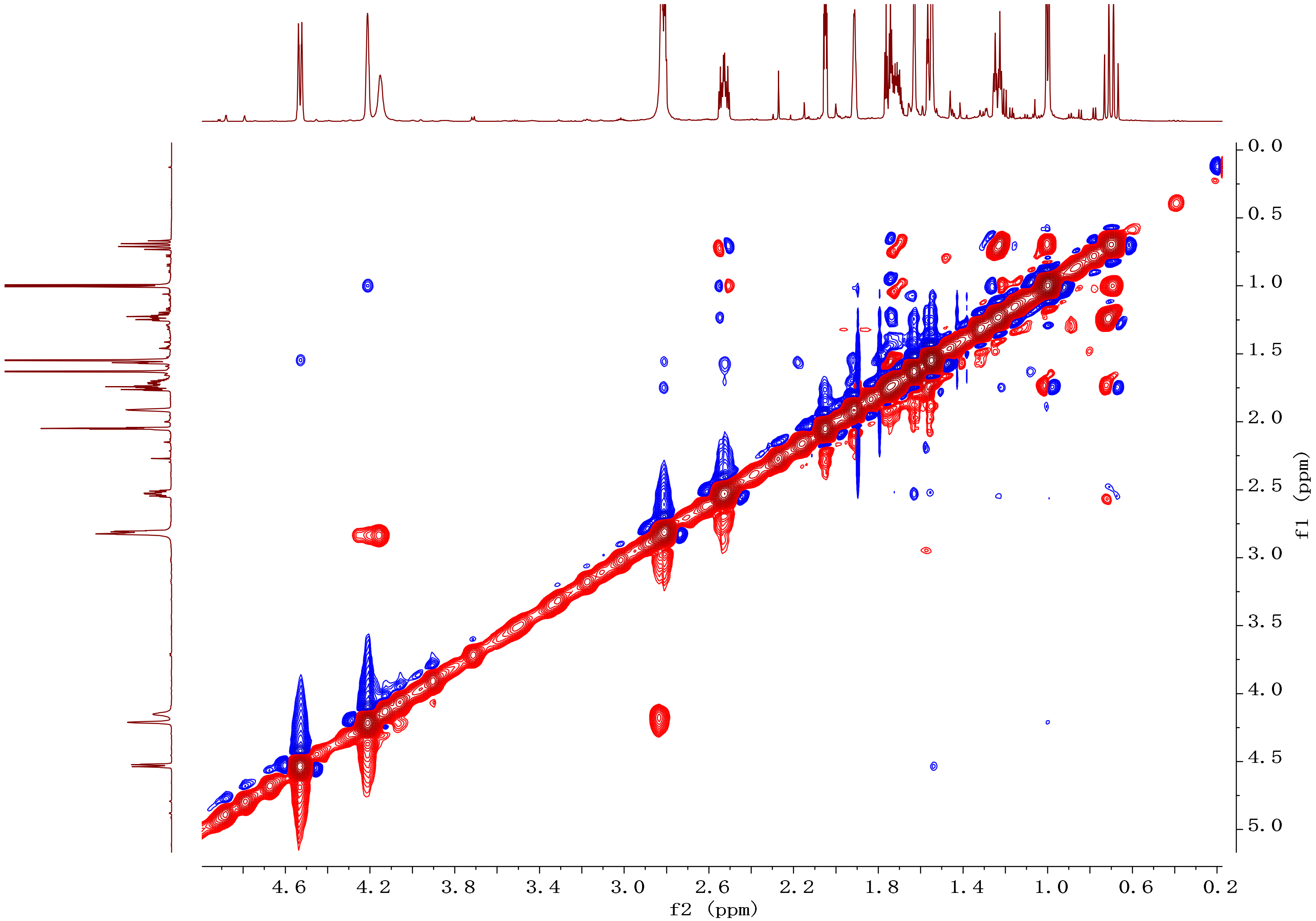


**Figure S11.** The NOESY spectrum of compound **1** in acetone-$d_6$

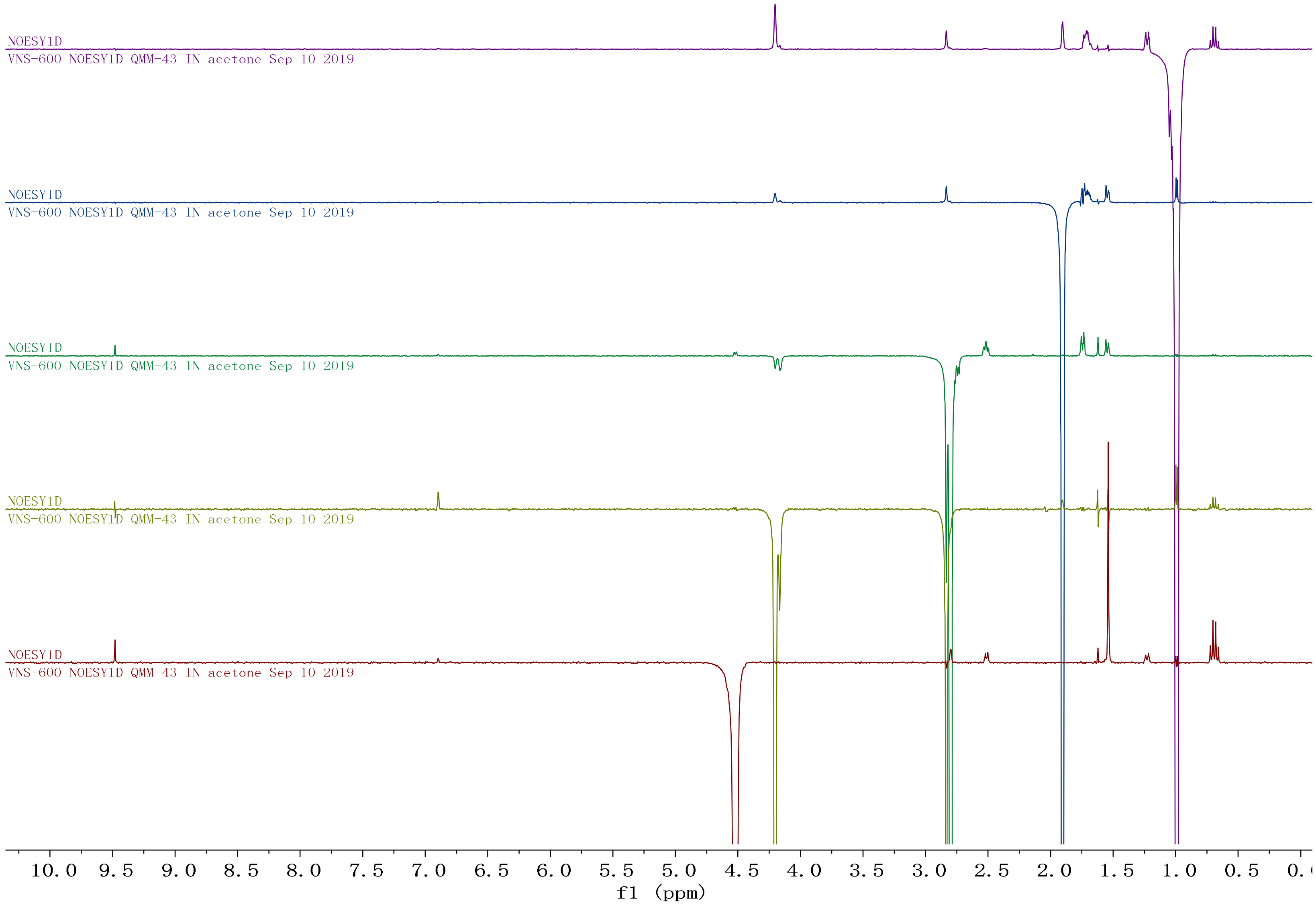


**Figure S12.** The 1D-NOE spectrum of compound **1** in acetone-$d_6$

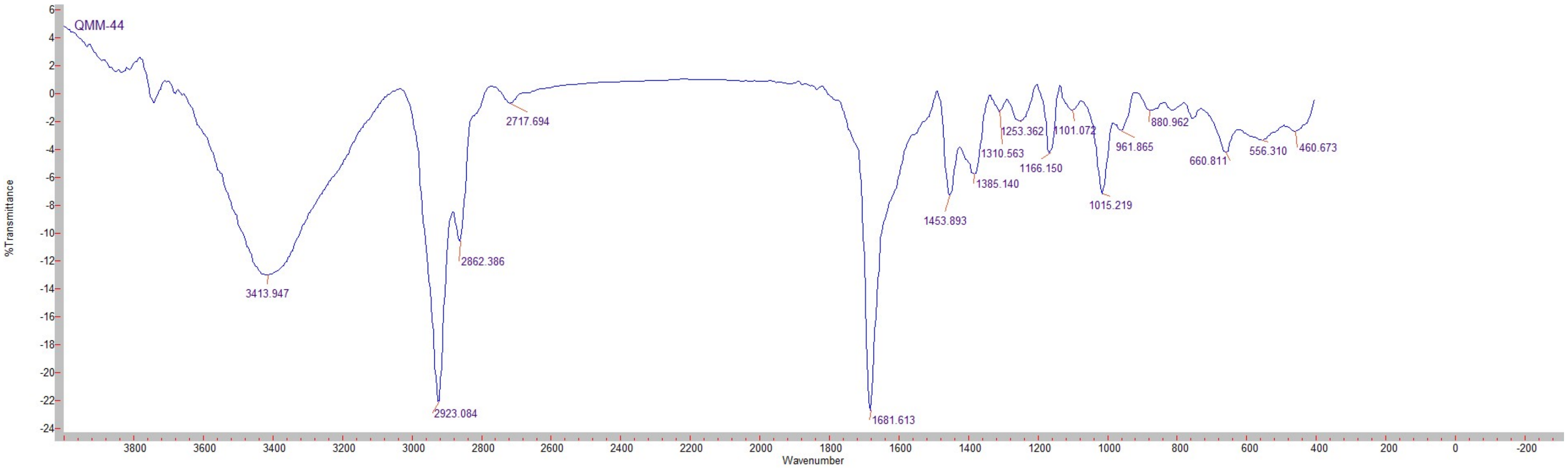


**Figure S13.** The IR spectrum of compound **2**

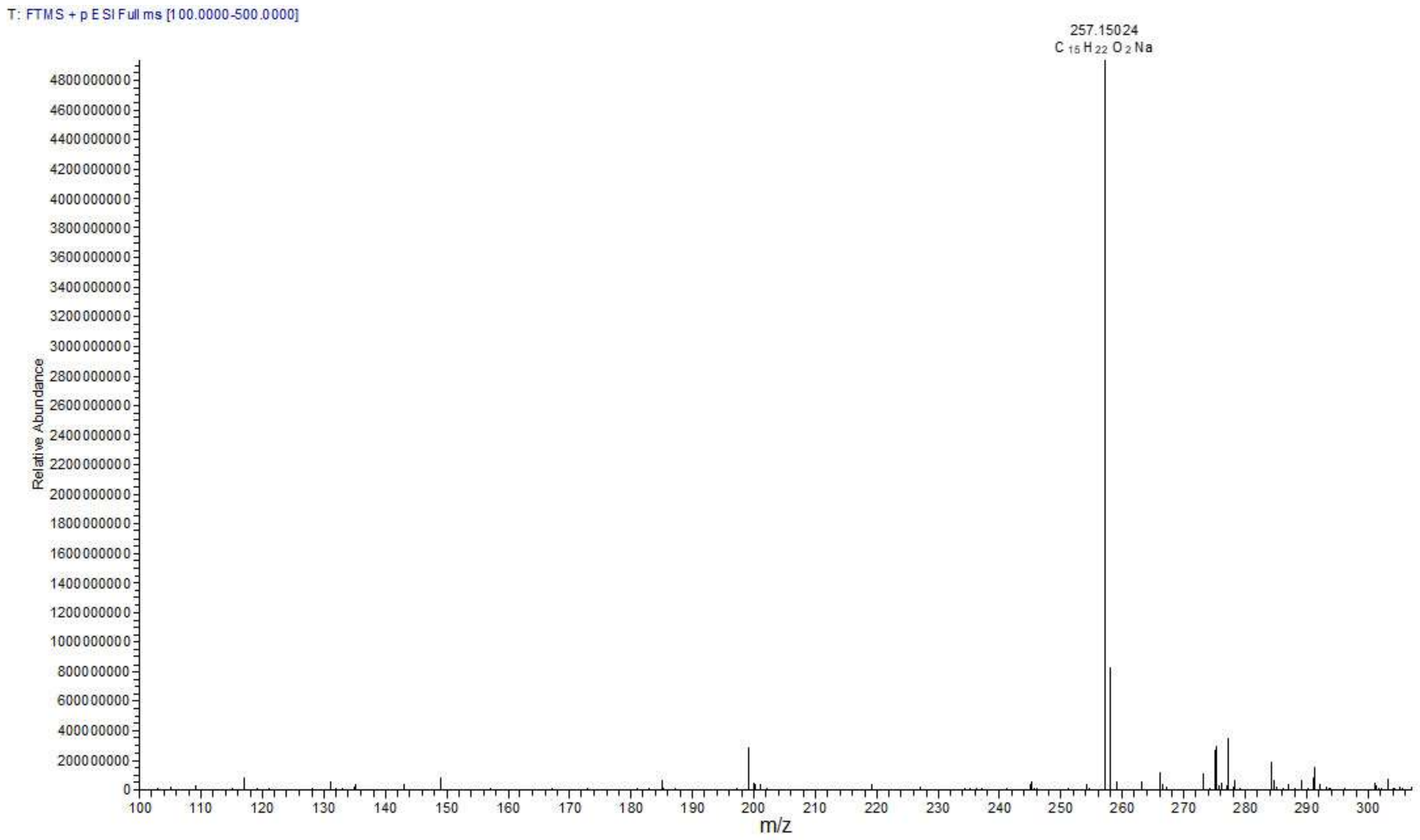


**Figure S14.** The (+)-HRESIMS spectroscopic data of compound **2**

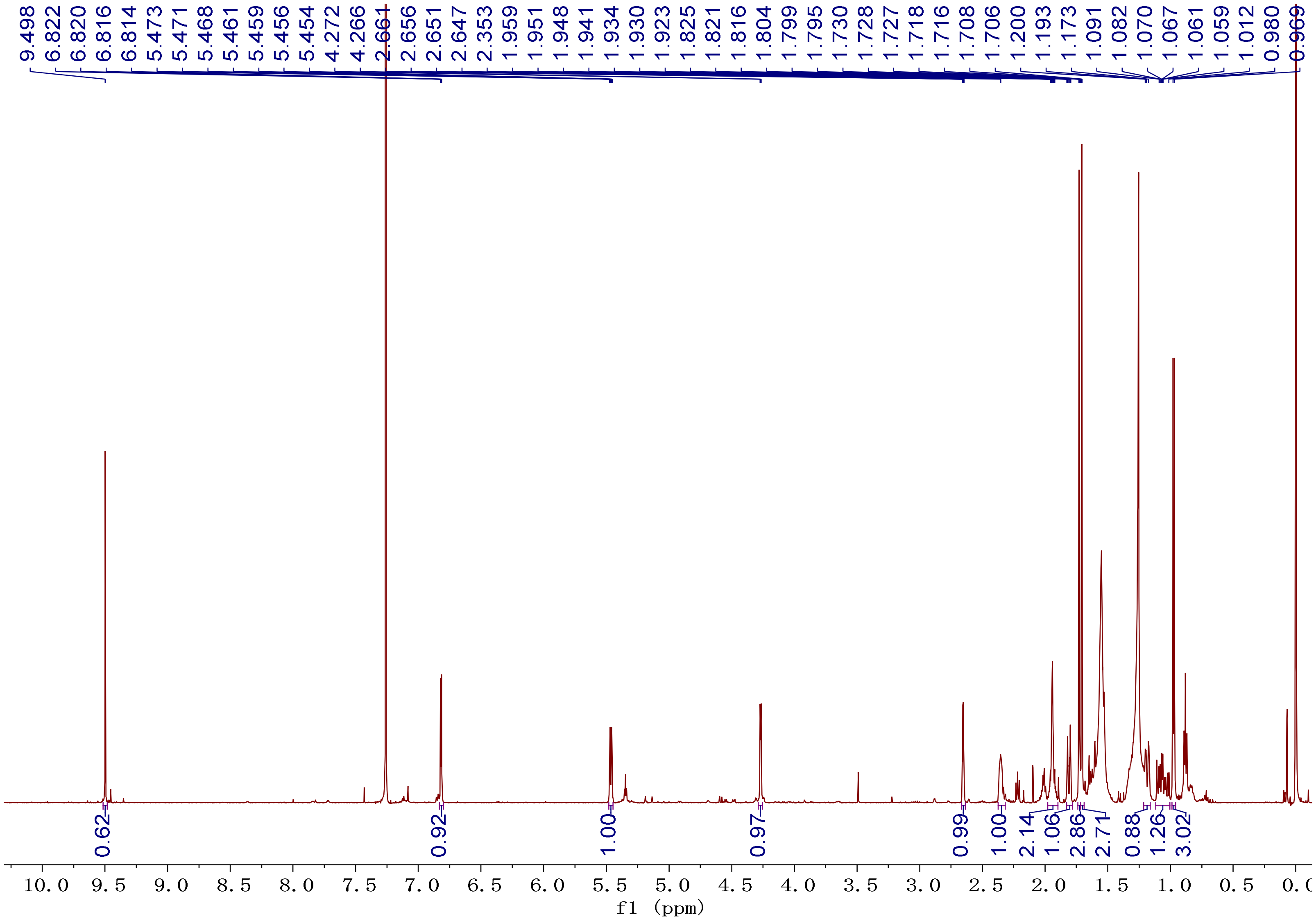


**Figure S15.** The $^{1}$H NMR spectrum of compound **2** in $CDCl_3$

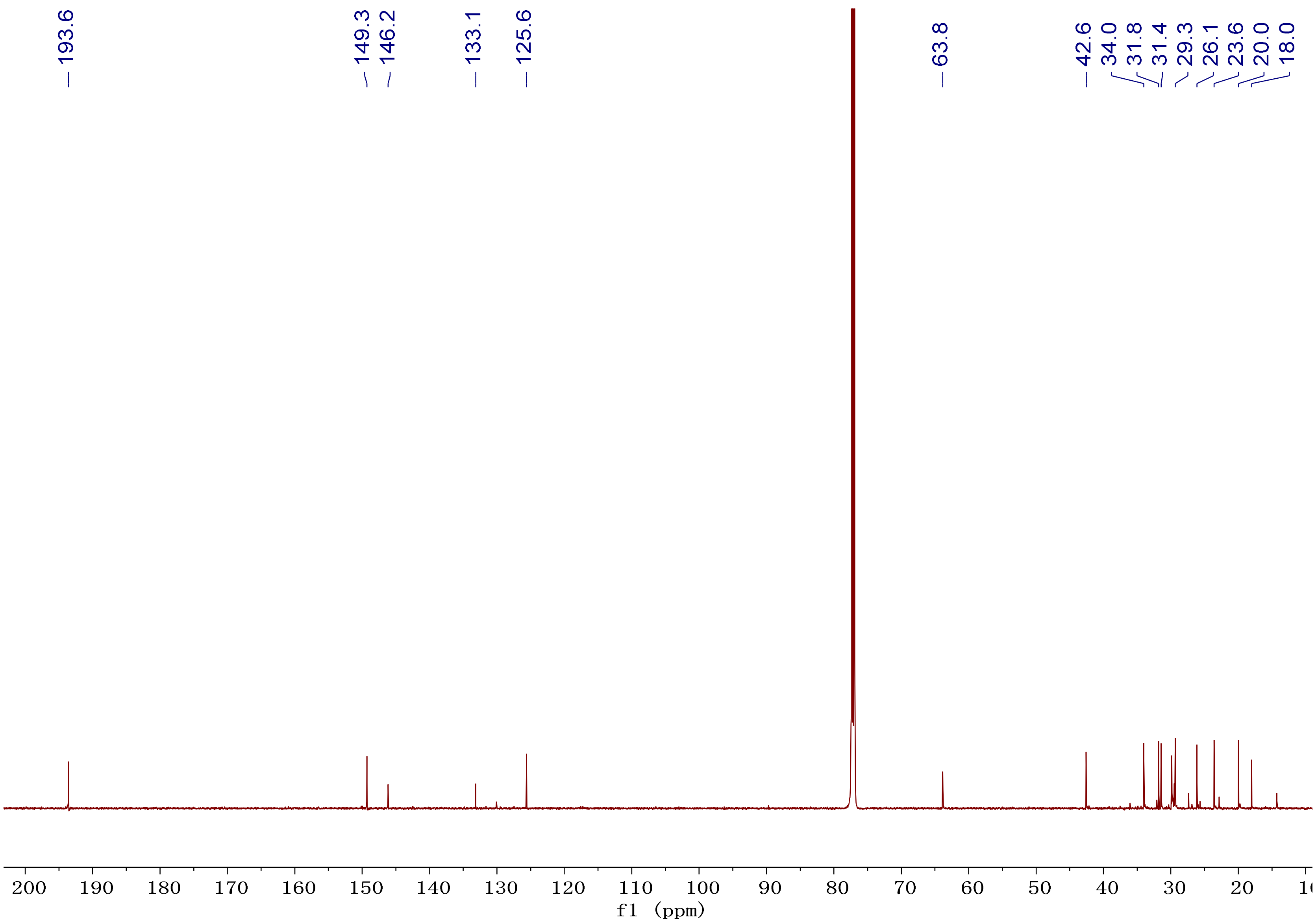


**Figure S16.** The $^{13}C$ NMR spectrum of compound **2** in $CDCl_3$

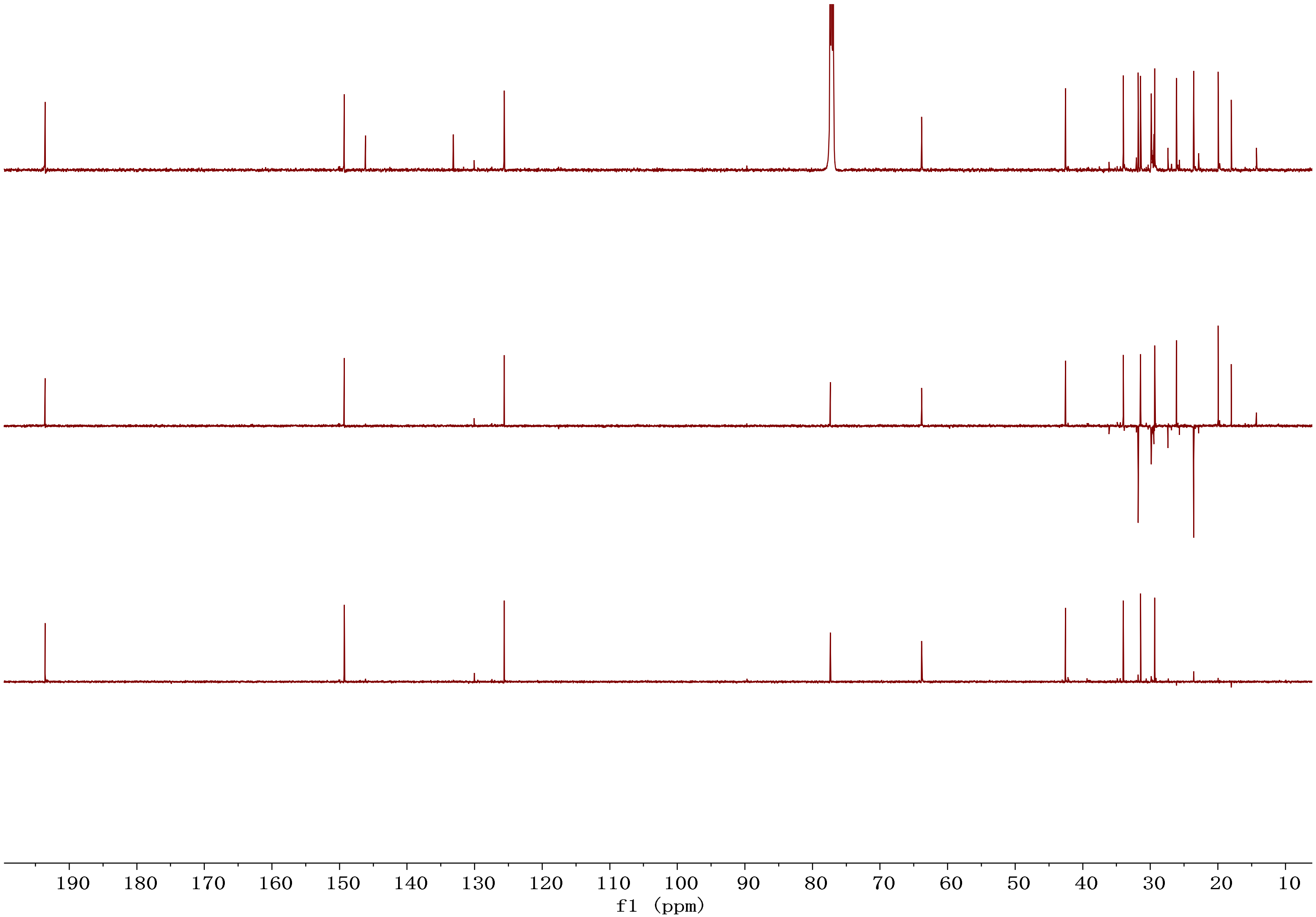


**Figure S17.** The DEPT spectrum of compound **2** in $CDCl_3$

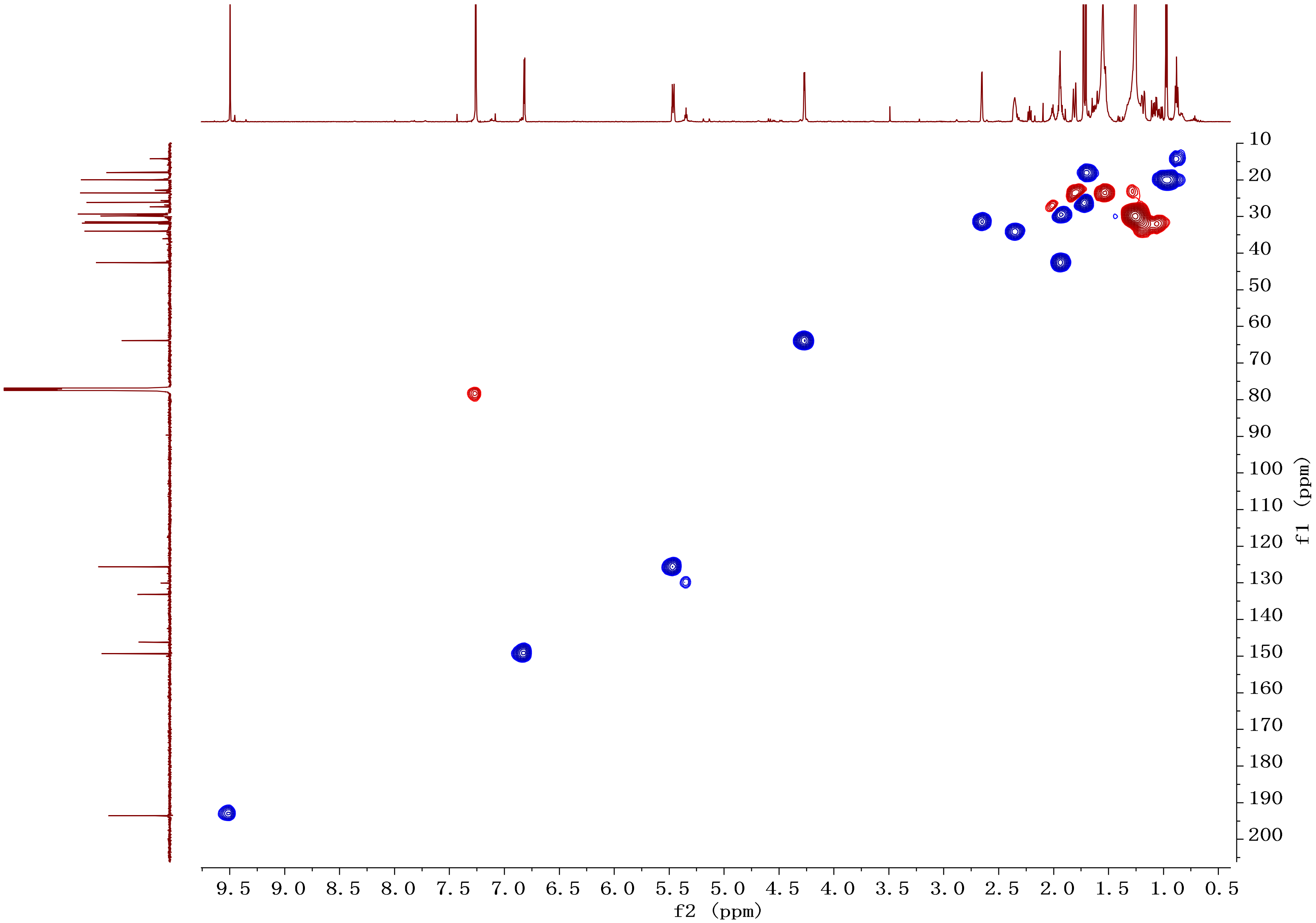


**Figure S18.** The HSQC spectrum of compound **2** in $CDCl_3$

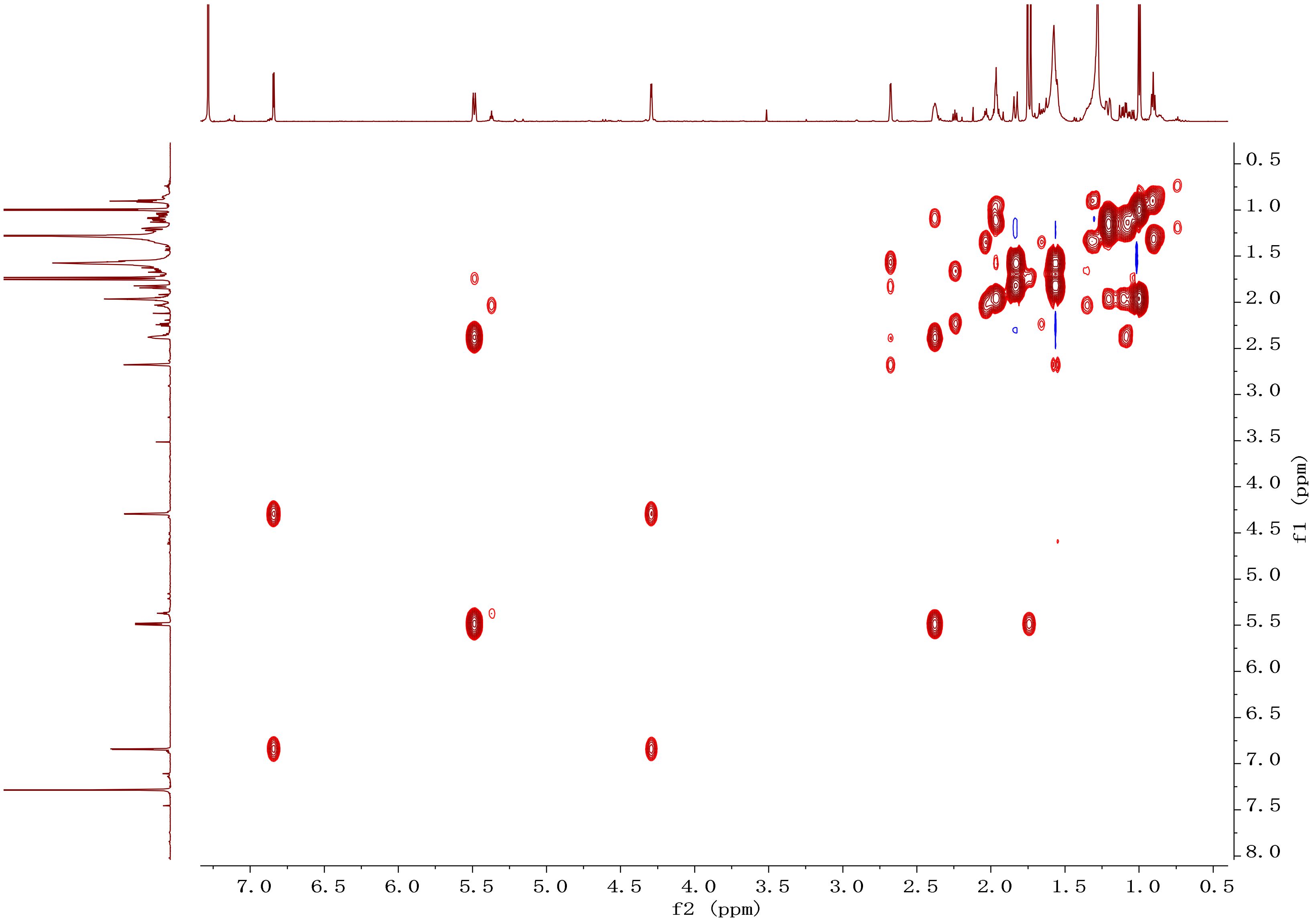


**Figure S19.** The $^{1}H$-$^{1}H$ gCOSY spectrum of compound **2** in $CDCl_3$

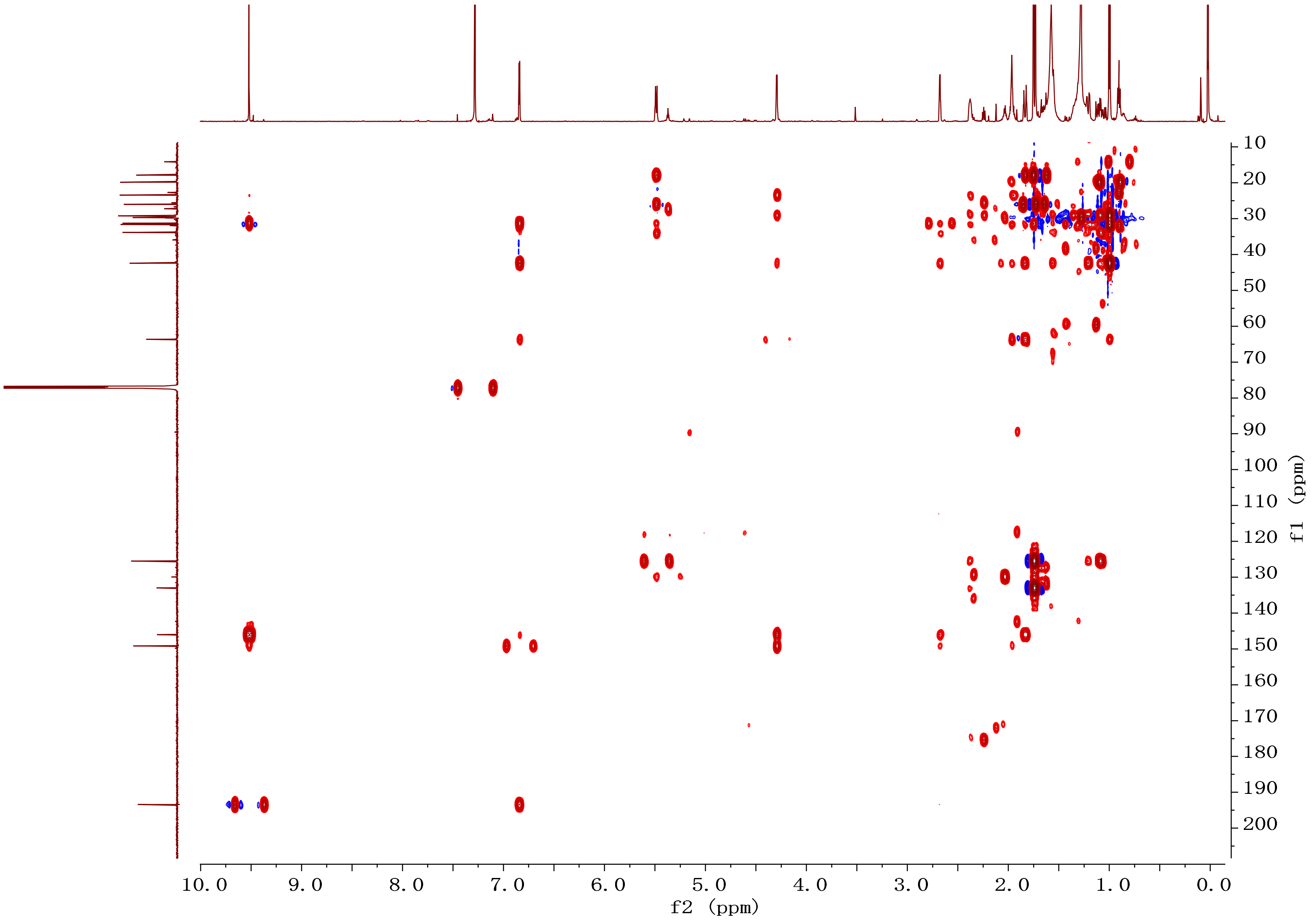


**Figure S20.** The HMBC spectrum of compound **2** in $CDCl_3$

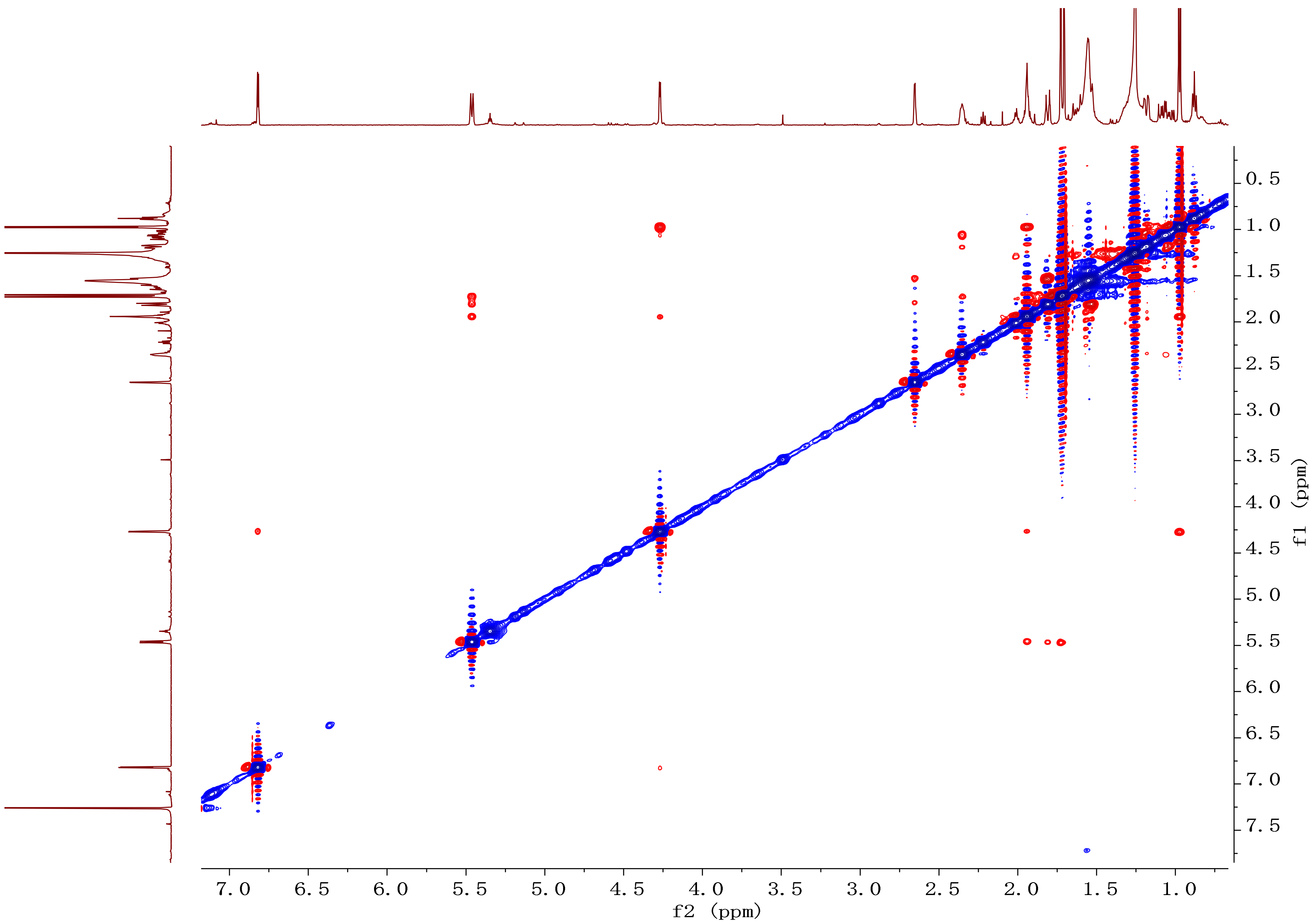


**Figure S21.** The NOESY spectrum of compound **2** in $CDCl_3$

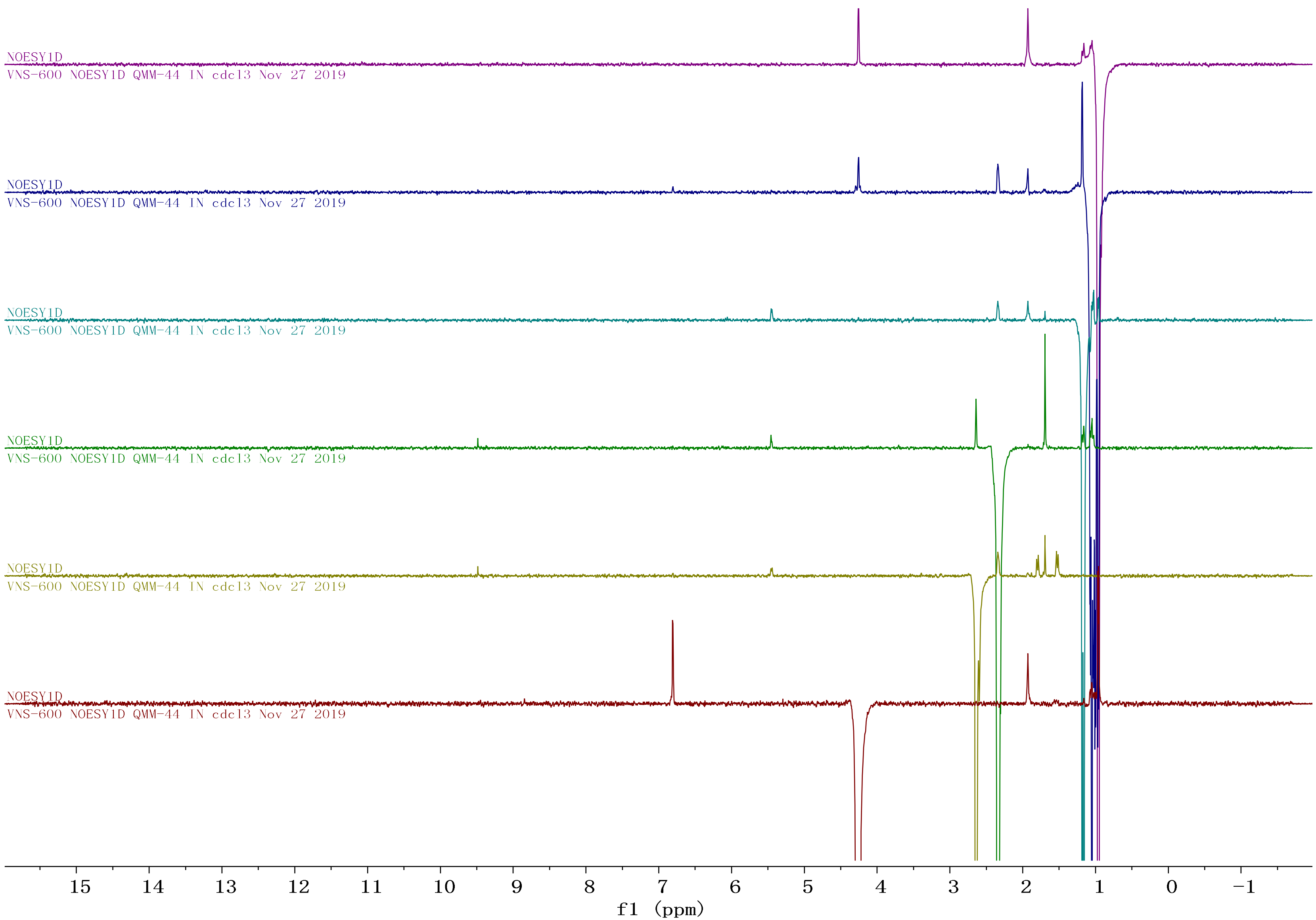


**Figure S22.** The 1D-NOE spectrum of compound **2** in $CDCl_3$
